# Obesogenic diet-induced gut barrier dysfunction and pathobiont expansion aggravate experimental colitis

**DOI:** 10.1371/journal.pone.0187515

**Published:** 2017-11-06

**Authors:** June-Chul Lee, Hae-Youn Lee, Tae Kang Kim, Min-Soo Kim, Young Mi Park, Jinyoung Kim, Kihyoun Park, Mi-Na Kweon, Seok-Hyung Kim, Jin-Woo Bae, Kyu Yeon Hur, Myung-Shik Lee

**Affiliations:** 1 Department of Medicine, Samsung Medical Center, Sungkyunkwan University School of Medicine, Seoul, Republic of Korea; 2 Severance Biomedical Science Institute and Department of Internal Medicine, Yonsei University College of Medicine, Seoul, Republic of Korea; 3 Department of Life and Nanopharmaceutical Sciences and Department of Biology, Kyung Hee University, Seoul, Republic of Korea; 4 Department of Health Sciences and Technology, SAIHST, Sungkyunkwan University, Seoul, Republic of Korea; 5 Department of Convergence Medicine, Asan Medical Center, University of Ulsan College of Medicine, Seoul, Republic of Korea; 6 Department of Pathology, Samsung Medical Center, Sungkyunkwan University School of Medicine, Seoul, Republic of Korea; 7 Department of Medicine, Division of Endocrinology and Metabolism, Samsung Medical Center, Sungkyunkwan University School of Medicine, Seoul, Republic of Korea; "INSERM", FRANCE

## Abstract

Consumption of a typical Western diet is a risk factor for several disorders. Metabolic syndrome is the most common disease associated with intake of excess fat. However, the incidence of inflammatory bowel disease is also greater in subjects consuming a Western diet, although the mechanism of this phenomenon is not clearly understood. We examined the morphological and functional changes of the intestine, the first site contacting dietary fat, in mice fed a high-fat diet (HFD) inducing obesity. Paneth cell area and production of antimicrobial peptides by Paneth cells were decreased in HFD-fed mice. Goblet cell number and secretion of mucin by goblet cells were also decreased, while intestinal permeability was increased in HFD-fed mice. HFD-fed mice were more susceptible to experimental colitis, and exhibited severe colonic inflammation, accompanied by the expansion of selected pathobionts such as *Atopobium* sp. and *Proteobacteria*. Fecal microbiota transplantation transferred the susceptibility to DSS-colitis, and antibiotic treatment abrogated colitis progression. These data suggest that an experimental HFD-induced Paneth cell dysfunction and subsequent intestinal dysbiosis characterized by pathobiont expansion can be predisposing factors to the development of inflammatory bowel disease.

## Introduction

Humans harbor approximately 10–100 trillion bacteria in the gut that are essential for human health [1,2]. However, bacterial translocation from the intestine into parenchymal tissues can break this symbiosis, resulting in pathological inflammatory consequences such as inflammatory bowel disease (IBD) or metabolic syndrome [3,4]. Despite the colossal number of intestinal bacteria, microbial translocation across this mucosal interface is a rare event, suggesting that the intestinal barrier utilizes a tightly regulated mechanism to control the translocation of commensal bacteria. Indeed, intestinal epithelium plays a protective role by secreting various antimicrobial factors [5,6]. Paneth cells, primarily located in the small intestine, secrete several mediators that serve to protect against enteric bacterial pathogens [7] and to establish the intestinal commensal microbiota [8]. Furthermore, other Paneth cell products function as vital trophic factors for stem cells in the small intestine [9], suggesting that aberrant Paneth cells can be a predisposing factor to the development of diverse infectious or inflammatory diseases and metabolic derangement. In addition to the AMPs produced by Paneth cells, mucin produced by goblet cells is important for the prevention of bacterial invasion across the intestine and protection of intestinal mucosa against diverse injuries [10].

The incidence of IBD has dramatically increased over the last few decades [11]. The development of IBD is associated with several susceptibility genes [4]. However, this disease is influenced by non-genetic external and internal environmental factors as well. As one external environmental factor, a Western diet has been epidemiologically linked to IBD development [12,13]. While metabolic syndrome is the most common disease associated with excessive intake of fat, the harmful effect of such a diet on the gut could also be substantial, because the gut can be directly affected by changes in diet composition. Enteric microbiota, considered an internal environmental factor, are involved in the development of IBD as well, and can be affected by dietary changes [14].

Here, we investigated high-fat diet (HFD)-induced changes of the gut as the first organ affected by consumption of a HFD and their impact on the development of experimental colitis. We found evidence that Paneth cell dysfunction and subsequent intestinal dysbiosis could be a predisposing factor to the development of IBD.

## Materials and methods

### Animals

Four-week-old male C57BL/6 mice (Jackson Laboratory) were fed normal chow diet (NCD) (13% fat, 25% protein, 62% carbohydrate; LabDiet 5053) or HFD (60% fat, 20% protein, 20% carbohydrate; Research Diets, D12492). *IL-6*-knockout mice were obtained from the Jackson Laboratory. Blood was withdrawn from retroorbital plexus under anesthesia by isoflurane (2%). At the end of the experiment, euthanasia was performed by deep anesthesia with isoflurane followed by cardiac puncture. All animal experiments were conducted in accordance with the Public Health Service Policy on Human Care and Use of Laboratory Animals. Mouse experiments were approved by the Institutional Animal Care and Use Committee of Samsung Medical Center Animal Facility, an Association for Assessment and Accreditation of Laboratory Animal Care (AAALAC) accredited unit.

### Histology, staining, and confocal microscopy

Mice intestines were flushed with phosphate-buffered saline (PBS) and fixed in 10% neural formalin overnight at room temperature. The paraffin-embedded specimens were cut into 5 μm sections and stained with hematoxylin and eosin (H&E) or periodic acid-Schiff (PAS)/Alcian blue. Paneth cells were stained with purple, and goblet cells blue with the PAS/Alcian blue method. For Paneth cell-specific antimicrobial peptide (AMP) staining, anti-procryptdin antibody (Ab) [15] generously provided by A. Ouellette (University of California, Irvine) was used. Paneth cell area or procryptdin content was quantified by measuring the eosinophilic granule area or the procryptdin-stained area per crypt area (μm^2^) using the positive pixel counting tool in the Aperio ImageScope software (Aperio Technologies, Inc.). For lysozyme staining, sections were stained with goat polyclonal anti-mouse lysozyme Ab (sc-27958, Santa Cruz Biotechnology), and further incubated with Alex 595-conjugated anti-goat Ab (Molecular Probes). For quantification of lysozyme content, thresholding was performed using ImageJ software (NIH), followed by densitometric quantification [16–19]. The specificity of the Ab was confirmed with mouse anti-lysozyme mAb (ab36362, Abcam) [20]. For Occludin, ZO-1, and RhoA staining, sections were incubated with rabbit polyclonal Abs against Occludin (40–4700, Invitrogen), ZO-1 (40–2200, Invitrogen), and RhoA (PAB18218, Abnova), respectively. To study Notch signaling, sections were stained with rabbit monoclonal Ab against the Notch intracellular domain (NICD) (ab52627, Abcam). To examine mTORC1 signaling, sections were stained with rabbit monoclonal Ab against phospho-S6 (4858, Cell Signaling). For mucin staining, intestinal segments were treated with methanol-Carnoy fixative [21], and dewaxed sections were stained with rabbit anti-mucin-2 mAb (sc-15334, Santa Cruz Biotechnology), and further incubated with Alex Fluor 488-conjuagated anti-rabbit Ab (Molecular Probes).

### Laser capture microdissection and quantitative reverse transcription polymerase chain reaction (RT-qPCR)

Total RNA was prepared from primary tissues or laser-dissected frozen sections of crypt area using TRIzol Reagent (Invitrogen) and an RNeasy Micro Kit (Qiagen), respectively [22,23]. cDNA was synthesized using Superscript II (Invitrogen) and oligo (dT)12–18 primers.

Quantitative RT-PCR primers used (5’ to 3’):

*Defcr-1*-forward, 5’-TCAAGAGGCTGCAAAGGAAGAGAAC-3’;
*Defcr-1*-reverse, 5’- TGGTCTCCATGTTCAGCGACAGC-3’;
*Defcr-4*-forward, 5’-CCAGGGGAAGATGACCAGGCTG-3’;
*Defcr-4*-reverse, 5’-TGCAGCGACGATTTCTACAAAGGC-3’;
*Defa-rs1c*-forward, 5’-CACCACCCAAGCTCCAAATACACAG-3’;
*Defa-rs1c*-reverse, 5’-ATCGTGAGGACCAAAAGCAAATGG-3’;
*Mucin-2*-forward, 5’-GCCTGTTTG ATAGCTGCTATGTGCC-3’;
*Mucin-2*-reverse, 5’-GTTCCGCCAGTCAATGCAGACAC-3’;
*Zo-1*-forward, 5’-TTTTTGACAGGGGGAGTGG-3’;
*Zo-1*-reverse, 5’-TGCTGCAGAGGTCAAAGT TCAAG-3’;
*Occludin*-forward 5’-ATGTCCGGCCGATGCTCTC-3’;
*Occludin*-reverse, 5’-TTTGGCTGCTCTTGGGTCTGTAT-3’;
*Rhoa*-forward, 5’- GCCAAAATGAAGCAGGAGCC-3’;
*Rhoa*-reverse, 5’- TACCCAAAAGCGCCAATCCT-3’;
*β-Actin*-forward, 5’-GCTGTGCTGTCCCTGTATGCCTCT-3’;
*β-Actin*-reverse, 5’-CTTCTCAGCTGTGGTGGTGAAGC-3’.
*Gapdh*-forward, 5’- AGGTCGGTGTGAACGGATTTG-3’
*Gapdh*-reverse, 5’- GGGGTCGTTGATGGCAACA-3’

### Crypt isolation, stimulation, and bactericidal activity assays

Small intestinal crypts were isolated according to published protocols [24]. In brief, the small intestinal lumen of adult mice was rinsed with ice-cold PBS, then segments were everted and shaken in Ca^++^ and Mg^++^-free PBS buffer containing 30 mM EDTA to detach crypts. Villi and crypts eluted at 5 min intervals were recovered by filtration through a 70 μm strainer followed by centrifugation at 700 × *g* at 4°C for 5 min, and crypts were identified using light microscopy. Crypt numbers were estimated via hemocytometry, and 2,000 crypts were resuspended in iPIPES buffer containing 10 μM carbachol (CCh) (Sigma) or 1 μg/mL lipopolysaccharides (LPS) (Sigma) and incubated at 37°C for 30 min. Bactericidal activity of crypt supernatants was assayed against 1 × 10^3^ CFU *S*. *typhimurium* CS015, as described previously [24].

### Cell death assay and flow cytometry

To assess cell death, crypts were dissociated into single cell suspensions by incubating the crypts in TrypLE Express (Invitrogen) containing 2,000 U/mL DNase (Sigma) for 30 min at 37°C. Dissociated cells were passed through a 20-μm cell strainer (Celltrix) and washed with PBS [9]. Cells were then stained using the Annexin V-fluorescein isothiocyanate (FITC) Apoptosis Detection Kit (eBioscience), and samples were acquired on a FACSCalibur cytometer equipped with CellQuest software (BD Biosciences) for analysis using FlowJo software (Tree Star). For specific quantification of Paneth cell death, dissociated crypt cells were stained with PE-conjugated anti-CD24 Ab (eBioscience) for 15 min at 37°C prior to staining using the Annexin V-FITC Apoptosis Detection Kit (eBioscience) for flow cytometric analysis [9]. To study crypt cell death *ex vivo*, oligonucleosome content in the cell lysate after 18 h incubation in culture medium was determined by ELISA with a commercial kit (Roche), according to the manufacturer’s instructions.

### Intestinal permeability

Age-matched mice were fed 0.6 mg/g body weight of 80 mg/mL FITC-dextran solution (Sigma), and peripheral blood was collected after 4 h [25]. Serum samples were diluted in equal volumes of PBS, and serum FITC-dextran concentrations were determined using a fluorescence spectrophotometer (GloMax-Multi Detection System, Promega) at an excitation wavelength of 490 nm and an emission wavelength of 540 nm. Serial dilutions of FITC-dextran in PBS were used as a standard curve.

### Dextran sodium sulfate (DSS)-induced colitis and flow cytometry

Acute colitis was induced by the administration of 3% (w/v) DSS (molecular mass 36–40 kDa; MP Biologicals) dissolved in sterile distilled water for 5 days. On day 7, mice were sacrificed and intraepithelial and lamina propria cells were isolated as previously described [26]. Briefly, intestinal IELs were isolated by longitudinal opening of the small intestine. 0.5 cm fragments were shaken for 30 min at 37°C in PBS supplemented with 10% FCS, 10 mM EDTA, 20 mM HEPES, 1 mM sodium pyruvate and 10 μg/ml Polymyxin B. Single cell suspension was further purified using 37.5% isotonic Percoll. After removal of the IEL fraction, the LP fraction was isolated by digestion with 0.4 mg/ml Liberase TL (Roche Applied Science) for a further 60 min at 37°C. Each subset of intestinal epithelial lymphocytes (IELs) and myeloid cells was analyzed via flow cytometry after staining with mAbs specific for TCRγδ, TCRαβ, CD8α, CD8β, CD3, CD11b, Ly6C, or F4/80 (eBiosciences).

### Histopathology and colitis scoring

On day 7 of DSS administration, the entire colon was excised, washed, fixed in 10% neutral buffer formalin and embedded in paraffin for H&E staining. The degree of inflammation (score 0–3) was evaluated by a pathologist in a blinded fashion: occasional inflammatory cells in the lamina propria = 0; increased number of inflammatory cells in the lamina propria = 1; confluence of inflammatory cells extending into the submucosa = 2; and transmural extension of the infiltrate = 3. The degree of tissue damage was evaluated in the same manner: no mucosal damage = 0; lymphoepithelial lesion = 1; surface mucosal erosion or focal ulceration = 2; and extensive mucosal damage and extension into deeper structures = 3. The sum of the inflammation and tissue damage scores was designated as the colitis score (0–6) [27].

### Colon organ culture

Organ culture of the colon was conducted using a modification of a previously published protocol [28,29]. Briefly, 1-cm segments of all 3 parts of the colon were washed in cold PBS supplemented with penicillin and streptomycin (Gibco). These segments were cultured in 24-well flat bottom culture plates (Falcon) in serum-free RPMI 1640 medium (Gibco) supplemented with penicillin and streptomycin. After 24 h, the supernatant was collected and stored at −20°C until measurement. Levels of IL-6, IL-1β, and TNF-α in the supernatant were determined using ELISA kits (R&D) according to the manufacturer’s instructions.

### Bacterial culture and colony-forming unit (CFU) assay

Liver tissue samples were collected in 5 mL of 3% thioglycolate solution and homogenized. Different dilutions of the resulting suspensions were plated on blood agar or brain heart infusion (BHI) agar, and incubated at 37°C for 48 h. Bacterial counts were determined by a colony-forming assay.

### 16S rRNA gene amplification and 454 pyrosequencing

Bacterial DNA was extracted from fecal samples, amplified, and sequenced [30]. Microbial community analysis based on 16S rRNA gene sequences was conducted using the QIIME software package version 1.8.0. In brief, the hypervariable regions (V1–V2) of 16S rRNA gene sequences were amplified using multiplex identifier adaptor primers (454 Life Sciences). The barcode primer set and approximately 10 ng of template DNA were added to a 50 μL PCR mix (Premix Ex Taq Hot Start Version, TaKaRa) for amplification under the following conditions: 94°C for 3 min, followed by 20 cycles at 94°C for 15 s, 55°C for 45 s, and 72°C for 1 min, and a final extension at 72°C for 8 min. Three independent PCR replicates for each sample were pooled, quantified using a spectrophotometer (NanoDrop ND-2000, Thermo Fisher Scientific), and combined in equimolar ratios. Pooled DNA was sequenced using a 454 GS FLX Titanium pyrosequencer (Roche 454 Life Sciences).

### Quantification of bacteria

Feces were obtained from mice fed either NCD or HFD. Collected samples were immediately frozen and stored at −80°C. Approximately 1 g of each sample was cut out from the sampled organs. Genomic DNA was then extracted from 0.5 g of each sample, according to the DNA extraction protocol [31], and proteinase K was added during cell lysis. Total bacterial DNA content was determined by quantitative PCR using the following 16S rRNA universal primers:

DPO-forward, 5’-AGAGTTTGATCMTGGCTCA-I-I-I-I-I-AACGCT-3;
DPO-reverse, 5’-CGCGGCTGCTGGCA-I-I-I-A-I-TTRGC-3 [32] or
EUBAC-forward, 5’-TCCTACGGGAGGCAGCAGT-3’;
EUBAC-reverse, 5’-GGACTACCAGGGTATCTAATCCTGTT-3’ as reported [3,33]. To quantify the change of microbial composition after fecal microbial transplantation (FMT), we used specific 16S rRNA gene primers for four phyla:
*Actinobacteria*-forward, 5’-GRDACYGCC GGGGTYAACT-3;
*Actinobacteria*-reverse, 5’-TCWGCGATTACTAGCGAC-3;
*Bacteroidetes*-forward, 5’-GCACGGGTGMGTAACRCGTACCCT;
*Bacteroidetes*-reverse, 5’-GTRTCTCAGTDCCARTGTGGG-3;
*Firmicutes*-forward, 5’-CAGCAGTAGGGA ATCTTC-3;
*Firmicutes*-reverse, 5’-ACCTACGTATTACCGCGG-3;
γ *Proteobcateria*-forward, 5’-CMATGCCGCGTGTGTGAA-3;
γ-*Proteobcateria*-reverse, 5’-ACTCCCCAGGCGGTCDACTTA-3 [34]. Quantitative PCR for total bacteria were carried out using a SYBR Premix Ex Taq (Takara) and ABI PRISM 7300 quantitative PCR System (Applied Biosystem). Data were analyzed by absolute quantification employing DNA standard curves obtained using genomic DNA of *E*.*coli* K12

### Analysis of 16S rRNA gene sequences and operational taxonomic units (OTUs)

Analysis of microbial community based on 16S rRNA gene sequences was conducted using the QIIME software package 1.8.0 [35] as previously described [36]. Briefly, raw sequences with ambiguous base calls, errors in the barcode and primer regions, average quality scores < 25, or lengths shorter than 200 base pairs were excluded. Subsequently, sequencing noise was removed using Denoiser, and the reverse primer sequences were also removed. Reference-based OTUs were clustered against the Greengenes core set at 97% sequence similarity using UCLUST algorithm. A representative sequence for each OTU was selected and aligned with the Greengenes core set using the PyNAST sequence aligner. Chimeric sequences were excluded using ChimeraSlayer program. A phylogenetic tree was constructed using the FastTree program. On the basis of an even-depth rarefied OTU table (640 sequences, the lowest number of sequences across the samples), the observed number of OTUs representing species richness was determined. Principal coordinate analysis (PCoA) was conducted based on UniFrac distances. Taxonomic assignment was performed by comparing the sequences with the Greengenes taxonomy strings using the naïve Bayesian RDP classifier, with a minimal confidence of 0.6.

### FMT

Mice were fed either an NCD or HFD for 15 weeks and then divided into 4 groups: NCD(NCD), NCD-fed mice transplanted with NCD microbiota; NCD(HFD), NCD-fed mice transplanted with HFD microbiota; HFD(NCD), HFD-fed mice transplanted with NCD microbiota; HFD(HFD), HFD-fed mice transplanted with HFD microbiota. Homogenized fecal pellets were transferred between groups as previously described [37–40]. Fecal sample were prepared by diluting 0.1 g feces in 1 ml PBS with 0.05% cysteine-HCl. After vortexing and settling by gravity for 2 min, 200 μl of the supernatant was administered by oral gavage 3 times a week for 3 weeks. Antibiotics were not administered before or during the FMT experiment. Three weeks after FMT, some mice were left untreated while others were treated with 3% DSS for 5 days and monitored for colitis progression.

### Antibiotic treatment

Mice were fed NCD or HFD and given drinking water containing antibiotic mixture or single antibiotics such as vancomycin or colistin for 8 weeks [37].

### Statistical analysis

All values are expressed as the means ± SEM. For all *in vitro* or *ex vivo* experiments, means ± SEM are representative of the results of more than 3 independent experiments that showed similar tendency. A student’s *t*-test was used to compare values between two groups. A one-way ANOVA was used to compare values among multiple groups. If an ANOVA test showed significant differences, the Duncan post-hoc test was used to compare two specific groups. If the Duncan test did not show a significant difference (*P* > 0.05), the two groups were labeled with the same letter over the respective bars. *P* values < 0.05 were considered statistically significant.

## Results

### HFD-dependent changes in Paneth cells

**Fig 1:**
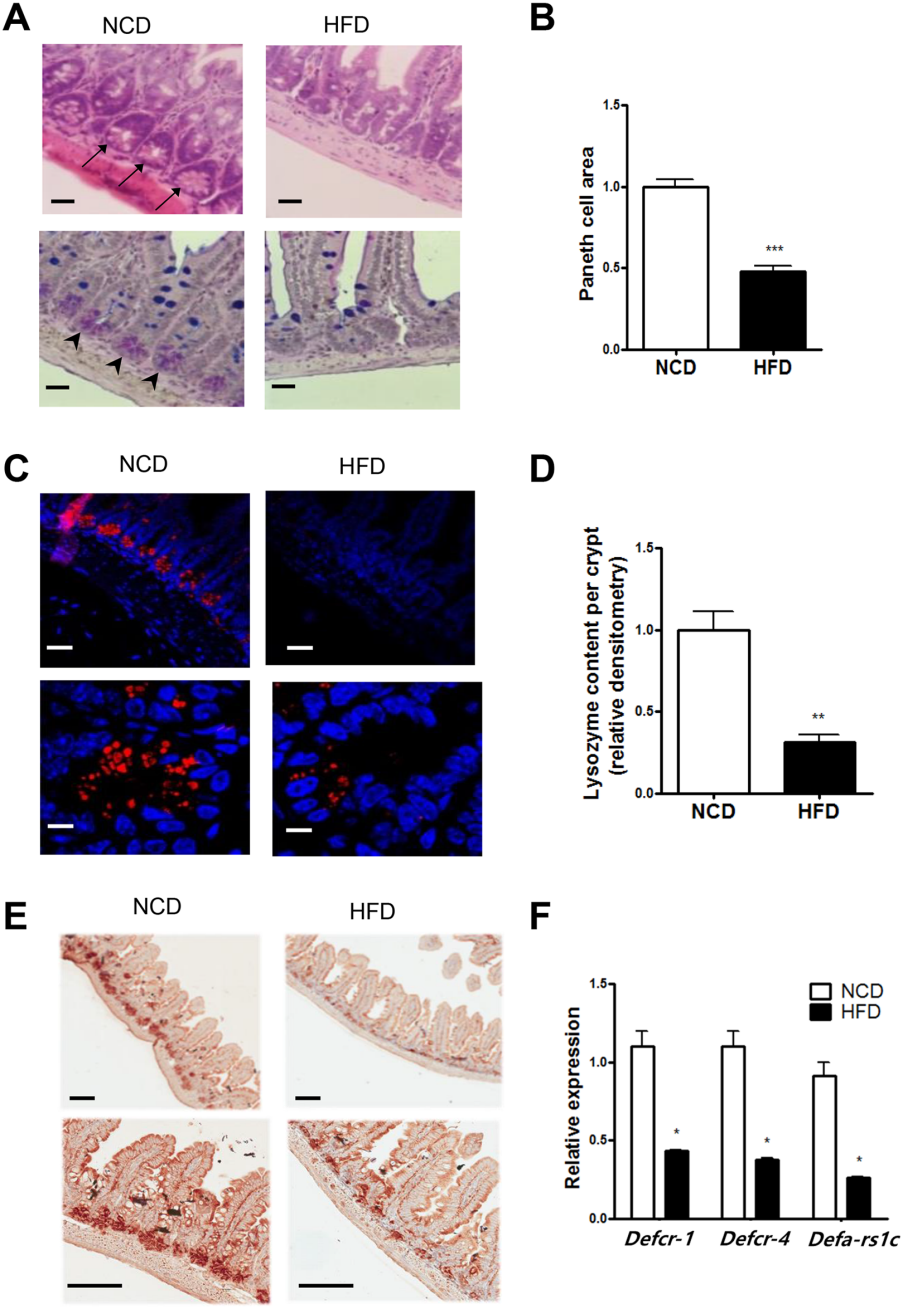
Decrease of Paneth cells in mice fed a high-fat diet (HFD). (A) Hematoxylin and eosin (H&E) staining of the small intestine showing typical eosinophilic granules of Paneth cells at the base of the crypts (upper row, arrows), and a Periodic acid-Schiff (PAS)/Alcian blue-stained section showing purple Paneth cell granules (lower row, arrow heads). Mice were fed either a normal chow diet (NCD) or a HFD for 15 weeks. (B) Quantification of the Paneth cell area was conducted as described in the Materials and methods (*n* = 5 each). (C) Confocal microscopic images of Paneth cells at the base of the crypts stained with anti-lysozyme Ab. (D) Quantification of lysozyme content per crypt was performed as described in the Materials and methods (*n* = 5 each). (E) Immunohistochemistry using anti-procryptdin Ab. (F) RT-qPCR analysis of antimicrobial peptides (AMPs) produced at the bottom of the crypts was performed using RNA isolated by laser capture microdissection (*n* = 5 each). Original magnification for all images is ×100, except the bottom row of (C) (×1,000) and the bottom row of (E) (×200). Scale bar, 100 μm. All values are expressed as the means ± SEM. A student’s *t*-test was used to compare values between two groups. *P < 0.05, **P < 0.01, and ***P < 0.001.

As one of the first organs contacting the HFD, we hypothesized that a HFD would disrupt the homeostasis and functions of the small intestine. H&E staining revealed notable morphological changes of Paneth cells, which control the microbial community in the gut by secreting AMPs, in the HFD-fed condition. The area of Paneth cells containing eosinophilic granules at the base of crypts was reduced in HFD-fed mice compared to NCD-fed mice (Fig 1A, upper row). PAS/Alcian blue staining also showed an apparent decrease in the number of purple-stained Paneth cell granules in HFD-fed mice (Fig 1A, lower row). Quantitative pixel counting revealed a significantly reduced Paneth area in HFD-fed mice compared to control mice (Fig 1B). Furthermore, immunofluorescence staining using anti-lysozyme Ab demonstrated a significant reduction of lysozyme content in each crypt in the distal ileum of HFD-fed mice (Fig 1C), which was consistent with the reduced Paneth cell granule content in these mice. Densitometric analysis also revealed a significantly reduced lysozyme content in crypts of HFD-fed mice compared to control mice (Fig 1D). Expression of procryptdin, an AMP exclusively produced by Paneth cells [15], was also notably decreased in the small intestine of HFD-fed mice (Fig 1E). When we employed laser capture microdissection to investigate the expression of other AMPs produced at the crypt bottom, the expression of multiple AMPs including *Defcr1*, *Defcr4*, and *Defa-rs1c* within crypts was significantly reduced in HFD-fed mice compared to control mice (Fig 1F). These results indicate that consumption of a HFD decreases Paneth cell area and downregulates the expression of multiple AMPs produced by Paneth cells, which may perturb the intestinal microbial community.

### Altered Paneth cell viability and function in HFD-fed mice

**Fig 2:**
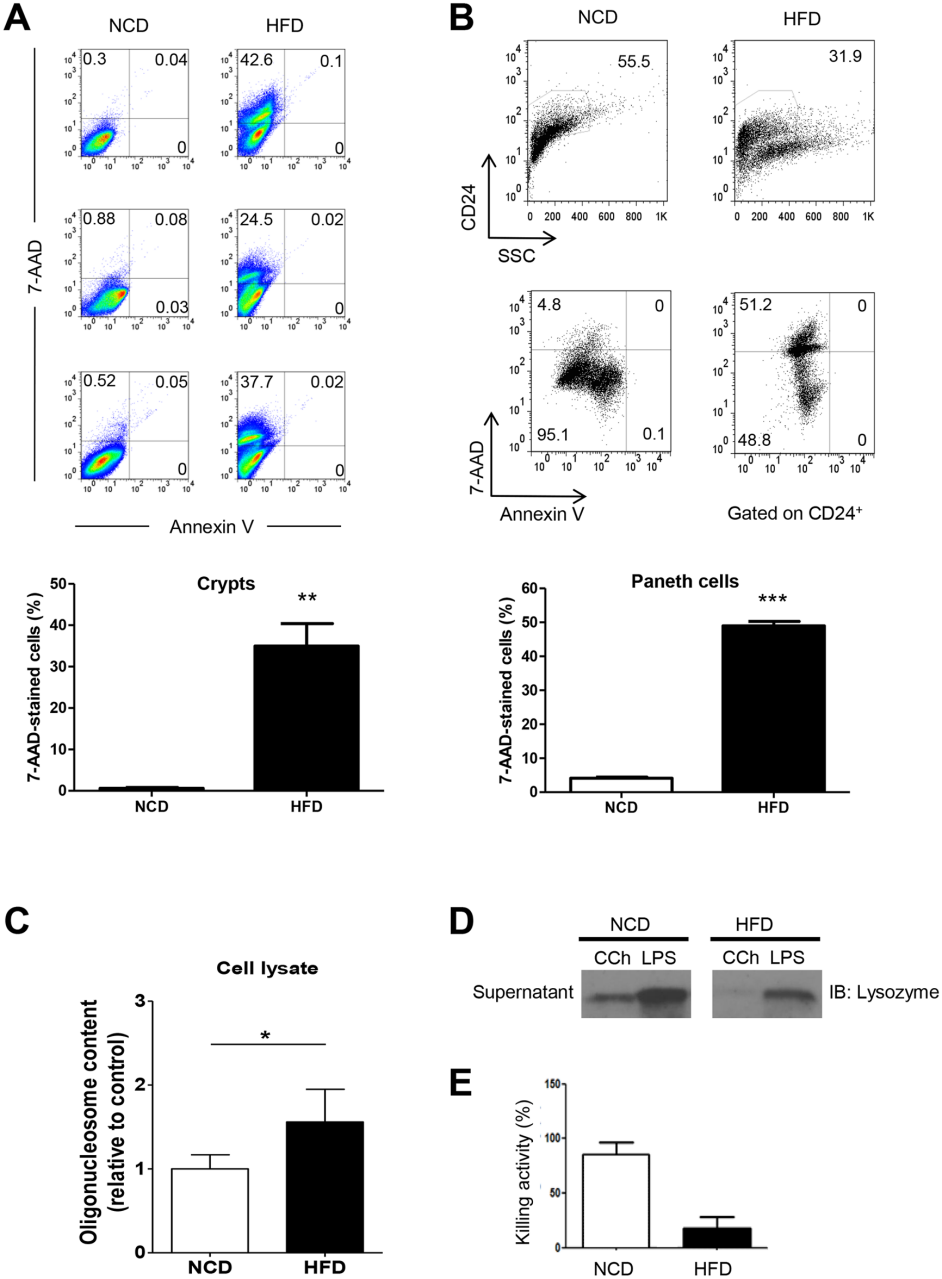
Paneth cell death and impaired Paneth cell function in mice fed a high-fat diet (HFD). (A) Crypt cell death *in vivo*. Crypts isolated from mice fed a normal chow diet (NCD) or a HFD were dissociated into single cells and stained using the Annexin V-FITC Apoptosis Detection Kit for flow cytometric analysis of cell death (upper panel). The percentage of 7-ADD-stained cells was compared between groups (lower panel). (B) CD24^+^ Paneth cell death in HFD-fed mice. Isolated crypts were dissociated into single cell suspensions, and CD24^+^ Paneth cell death was determined using an Annexin V-FITC Apoptosis Detection Kit for flow cytometric analysis of cell death gated on CD24 (upper panel). The percentage of 7-ADD-stained cells among CD24^+^ Paneth cells was compared between groups (lower panel). (C) Crypt cell death *ex vivo*. Crypts isolated from NCD-or HFD-fed mice were cultured *ex vivo* for 18 h, and DNA oligonucleosome contents in the cell lysate were measured (n = 3). (D) Crypts were stimulated with carbachol (CCh) for 30 min, and the supernatants were subjected to an immunoblot (IB) assay using anti-lysozyme Ab. (E) Bactericidal activity of intestinal crypt supernatant against *Salmonella typhimurium*. Data are expressed as the percentage of killed bacteria relative to unexposed bacteria (*n* = 3 each). All values are expressed as the means ± SEM. A student’s *t*-test was used to compare values between two groups. *P < 0.05, **P < 0.01, ***P < 0.001.

We next examined the mechanism causing decreased Paneth cell mass in HFD-fed mice. A cell death assay using Annexin V/7-AAD staining revealed pronounced death of intestinal crypt cells in HFD-fed mice compared to NCD-fed mice (Fig 2A). When we employed anti-CD24 mAb to identify death of Paneth cells among crypt cells [9,14], we observed a decrease in the total number of CD24^+^ Paneth cells and an increase in the number of 7-AAD-stained CD24^+^ cells in HFD-fed mice compared to NCD-fed mice (Fig 2B), suggesting that Paneth cell death due to HFD is a cause of decreased Paneth cell mass in mice fed HFD. To further investigate the viability of crypt cells in HFD-fed mice, we cultured crypt cells *ex vivo* without any treatment and monitored cell death. DNA oligonucleosome content in the cell lysate was significantly increased after *ex vivo* culture of crypt cells from HFD-fed mice for 18 h compared to those from NCD-fed mice (Fig 2C), suggesting reduced viability of crypt cells from HFD-fed mice.

We also examined the functional consequences of Paneth cell changes in HFD-fed mice. Western blot analysis of the culture supernatant after stimulation of isolated crypts with Paneth cell secretagogues, such as LPS or CCh [41], showed significantly reduced lysozyme release from the crypts of HFD-fed mice compared to those of NCD-fed mice (Fig 2D). Using a bacterial killing assay that employs *Salmonella typhimurium* [42], we found that the bactericidal killing activity of the crypt supernatant from HFD-fed mice was significantly reduced compared to control mice (Fig 2E), suggesting that consumption of a HFD impairs Paneth cell function.

### HFD-dependent changes in goblet cells

**Fig 3:**
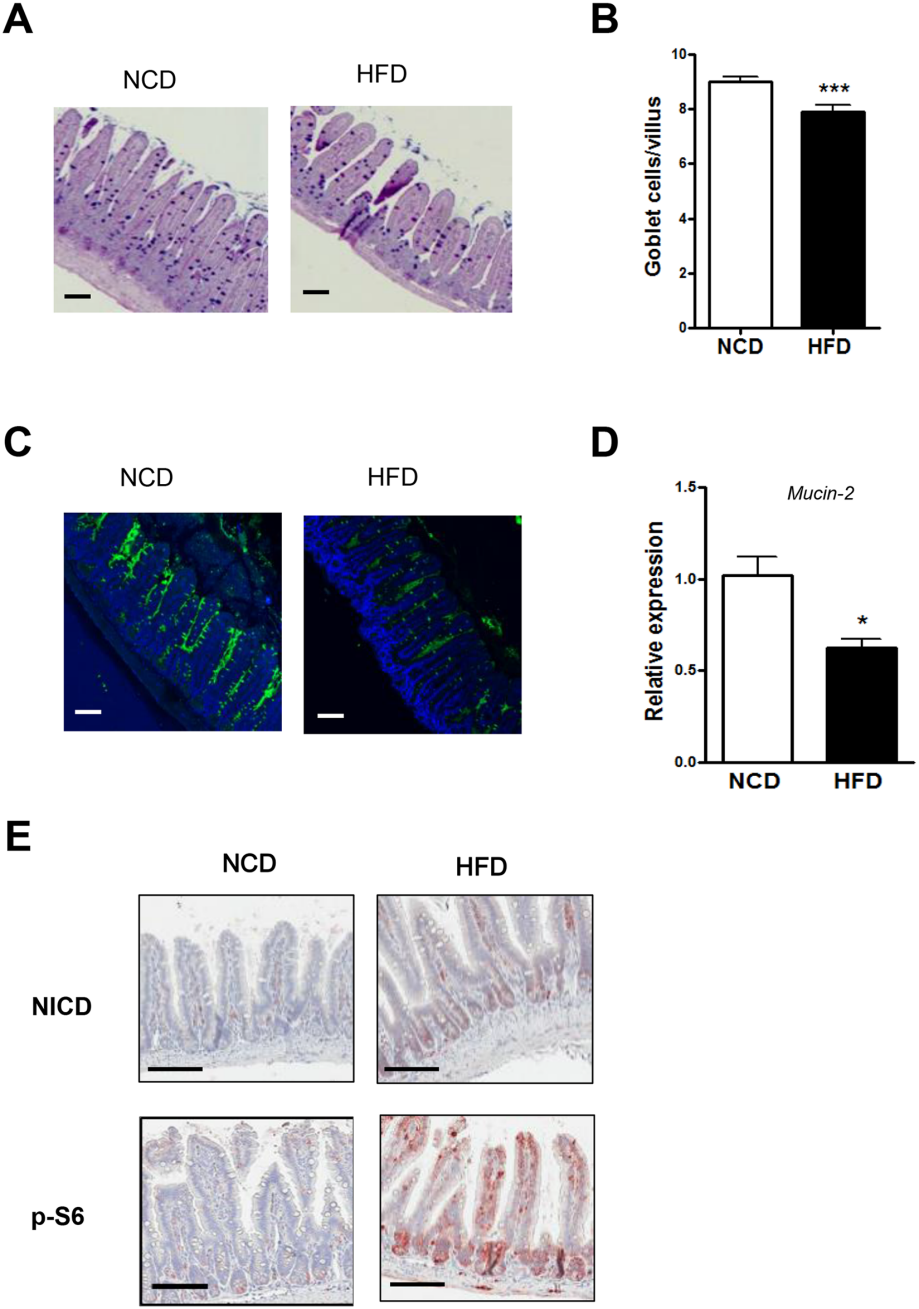
Decrease of goblet cells in mice fed a high-fat diet (HFD). (A and B) Changes in goblet cells in HFD-fed mice. Representative PAS/Alcian blue-stained sections of the distal ileum from NCD- or HFD-fed mice showing goblet cells (blue) (A) and quantification of goblet cell number (n = 5 each) (B). (C and D) Changes in Mucin-2 expression. Confocal microscopic images of the distal ileum following immunofluorescence staining using anti-Mucin-2 mAb (C) and relative expression of *Mucin-2* determined by RT-qPCR (*n* = 5 each) (D). (E) Immunohistochemistry using anti-Notch intracellular domain (NICD) and phospho-S6 Abs. Original magnification for all images is ×100, except (E) (×200). Scale bar, 100 μm. All values are expressed as the means ± SEM. A student’s *t*-test was used to compare values between two groups. *P < 0.05 and ***P < 0.001.

Because mucin is at the frontline of the physical barrier preventing access of enteric bacteria to intestinal epithelium [43], we investigated whether the number of goblet cells producing mucin is affected by consumption of a HFD. PAS/Alcian blue staining showed that the number of blue-stained goblet cells was significantly reduced in the small intestine of HFD-fed mice (Fig 3A and 3B), consistent with a previous report [30]. Confocal assays using anti-mucin-2 monoclonal Ab (mAb) also showed a reduced mucin content in the small intestine of HFD-fed mice (Fig 3C). Consistently, RT-qPCR revealed reduced *Mucin-2* expression in gut epithelium of HFD-fed mice compared to NCD-fed mice (Fig 3D). To investigate the potential mechanism underlying the reduced *Mucin-2* expression in HFD-fed mice, we studied the Notch pathway, which can control intestinal goblet cell differentiation or mucin production and can be influenced by nutritional signaling [44–46]. Indeed, the expression of the Notch intracellular domain (NICD), representing Notch activation, was markedly increased in the small intestine of mice fed HFD compared to mice fed NCD (Fig 3E, upper row), suggesting that Notch activation plays a role in the reduced expression of Mucin-2 [46]. We also studied the mTORC1 pathway, which can influence Notch signaling in association with altered nutrient status [46]. The expression of phospho-S6 representing mTORC1 activity was markedly upregulated in the small intestine of HFD-fed mice (Fig 3E, lower row), suggesting that mTORC1 activation associated with HFD feeding leads to Notch activation and subsequent downregulation of Mucin-2 expression [45,46].

### Impaired gut barrier function in HFD-fed mice

**Fig 4:**
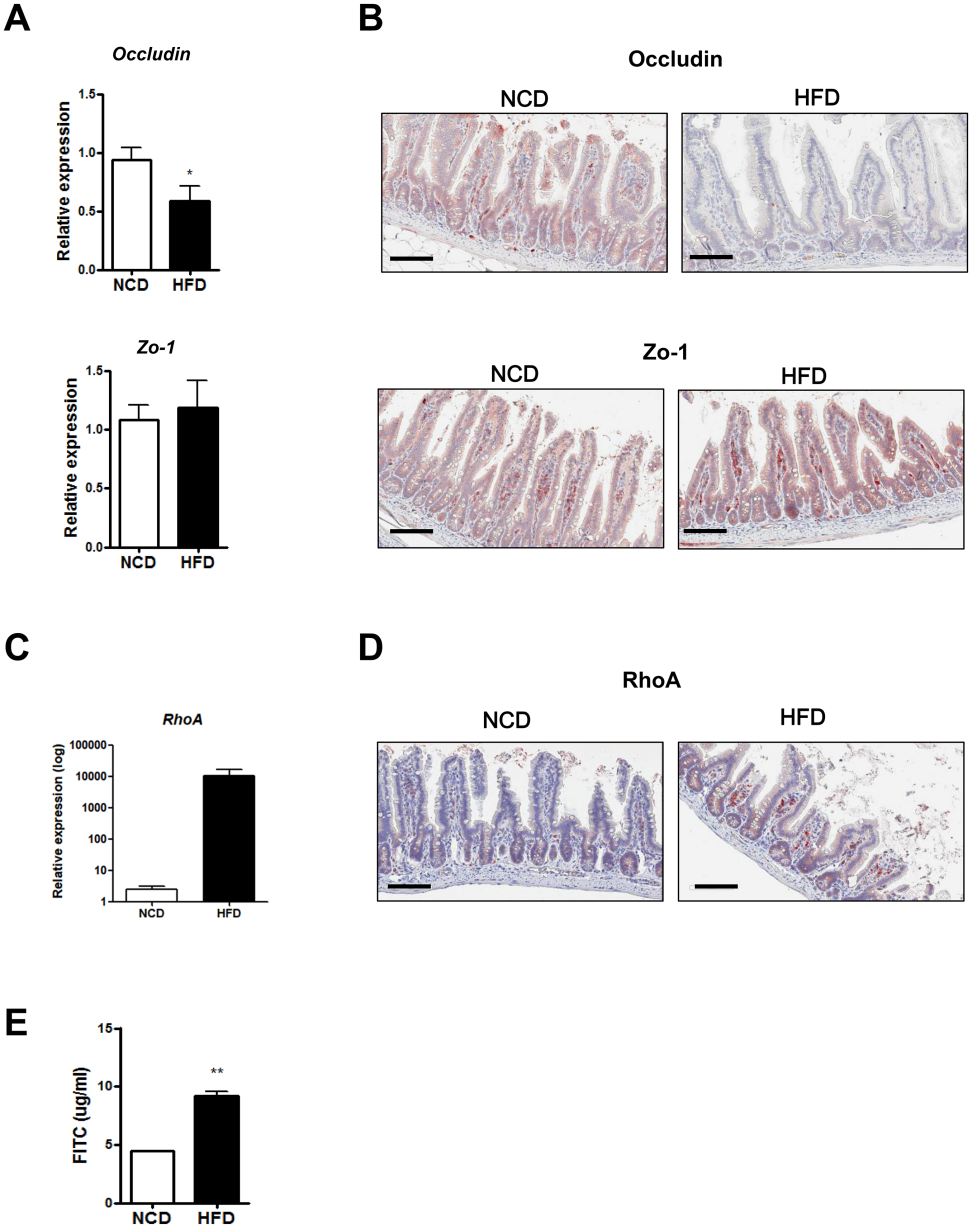
Disruption of the gut barrier in high-fat diet (HFD)-fed mice. (A) RT-qPCR analysis of *Occludin* and *Zo-1* using RNA from the distal ileum of normal chow diet (NCD)- or HFD-fed mice (*n* = 5 each). (B) Representative immunohistochemistry of distal ileal sections using anti-Occludin or -Zo-1 mAb. (C and D) RhoA expression in the intestine of mice fed NCD or HFD. mRNA expression (C) and representative immunohistochemistry (D) of RhoA in the small intestine. (E) Serum levels of FITC 4 h after oral FITC-dextran administration to NCD- or HFD-fed mice by oral gavage (*n* = 5 each). Original magnification ×200 for all images. Scale bar, 100 μm. All values are expressed as the means ± SEM. A student’s *t*-test was used to compare values between two groups. *P < 0.05, **P < 0.01.

We next examined the integrity of intestinal epithelium, an important physical barrier in addition to mucin. RT-qPCR showed that mRNA expression of *Occludin*, which encodes a tight junction protein, was significantly reduced in the ilea of HFD-fed mice compared to NCD-fed mice (Fig 4A, upper), consistent with a previous report [47]. In contrast, mRNA expression of *Zo-1*, encoding another tight junction protein, was similar between the two groups (Fig 4A, lower). Consistent with these data, immunohistochemistry revealed markedly reduced expression of Occludin in the intestinal epithelium of HFD-fed mice compared to NCD-fed mice (Fig 4B, upper row), while Zo-1 expression was unchanged (Fig 4B, lower row). We also examined the expression of RhoA, which regulates tight junction assembly and actin organization in the small intestine [48,49]. The expression of *RhoA* determined by RT-qPCR was significantly upregulated in the intestine of HFD-fed mice compared with NCD-fed mice (Fig 4C). Immunohistochemistry also showed a marked increase in expression of RhoA in the intestine of HFD-fed mice compared with NCD-fed mice (Fig 4D), suggesting that RhoA activation may play a role in the impairment of gut barrier integrity by HFD feeding.

We further investigated the barrier function of absorptive epithelia by measuring circulating FITC-dextran 4 h after an oral gavage with FITC-dextran. We found that HFD-fed mice exhibited a significantly higher serum FITC fluorescence compared to NCD-fed mice, suggesting impaired gut barrier function in HFD-fed mice (Fig 4E).

### Aggravated experimental colitis in HFD-fed mice

**Fig 5:**
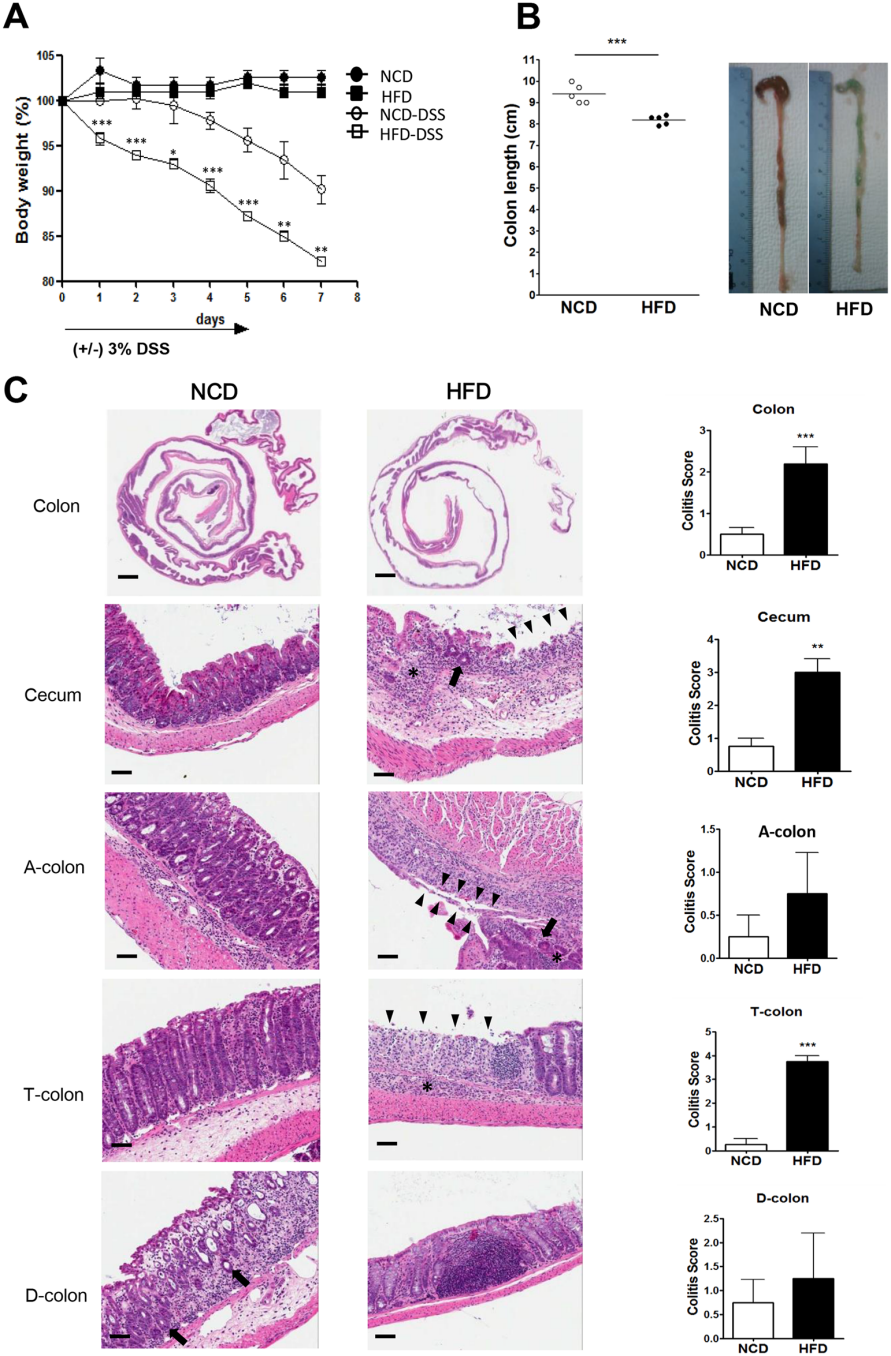
Aggravated experimental colitis in high-fat diet (HFD)-fed mice. (A) Mice fed normal chow diet (NCD) or HFD for 15 weeks were either untreated or treated with 3% dextran sodium sulfate (DSS) for 5 days, and monitored for colitis progression. Body weight is presented as the percentage of the initial weight. (B) Colon lengths measured on day 7 of DSS administration (left). Representative images of the colon are shown (right). (C) Representative H&E sections of the cecum, the ascending colon (A-), transverse colon (T-), and descending (D-) colon on day 7 of DSS administration to NCD-fed (left column) or HFD-fed (middle column) mice (arrowheads, ulceration; asterisks, inflammation; arrows, regeneration of crypts). Colitis scoring on day 7 of DSS administration (right column). Original magnification for all images is ×100, except the top row of (C) (×10). Scale bar, 100 μm. All values are expressed as the means ± SEM. A student’s *t*-test was used to compare values between two groups. *P < 0.05, **P < 0.01, ***P < 0.001 (*n* = 5 each).

Because Paneth cell dysfunction is important in the pathogenesis of IBD [5,41] and a Western diet is associated with an increased incidence of IBD [12], we examined whether HFD-induced Paneth cell dysfunction aggravates DSS-induced colitis, a model used to study the pathogenesis of IBD [50]. Mice fed NCD or HFD for 15 weeks were either untreated or treated with 3% DSS for 5 days, and monitored for colitis progression. Percent body weight loss after DSS treatment was significantly higher in HFD-fed mice compared to NCD-fed mice, while there was no difference in the percent body weight between HFD-fed and NCD-fed mice without DSS treatment (Fig 5A). Furthermore, the colons of HFD-fed DSS-treated mice were on average 20% shorter than those of NCD-fed DSS-treated mice (Fig 5B). Microscopic examination revealed more severe histological changes in the colons of HFD-fed DSS-treated mice compared to NCD-fed DSS-treated mice. Specifically, extensive ulceration and inflammation accompanied by crypt regeneration were frequently observed in the colons of HFD-fed DSS-treated mice, resulting in significantly increased colitis scores in the various segments of the colon (Fig 5C).

**Fig 6:**
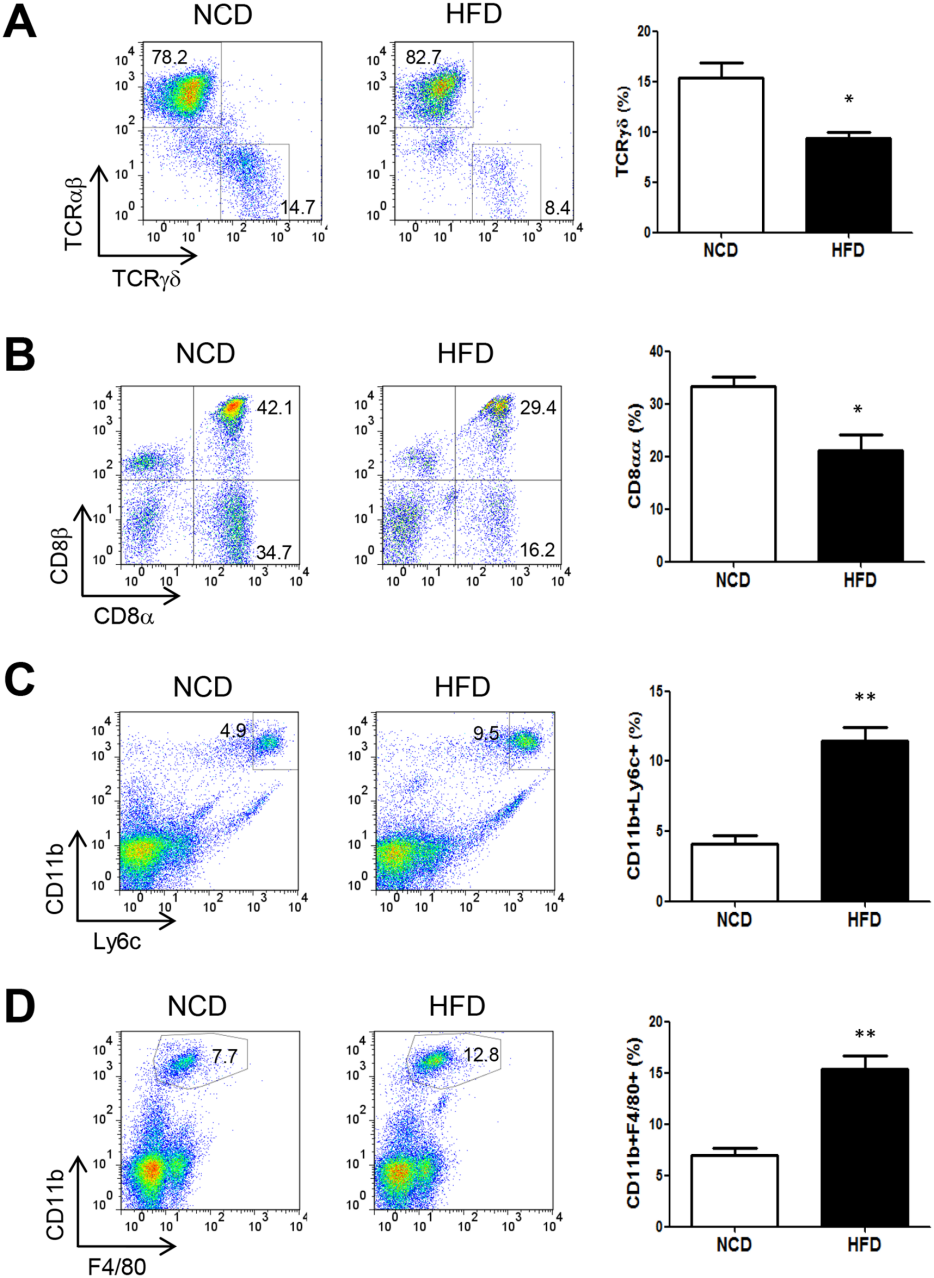
Flow cytometric analysis of cells in the colonic epithelium of high-fat diet (HFD)-fed mice treated with dextran sodium sulfate (DSS). (A and B) Proportions of T cell subsets. Proportions of TCRγδ and TCRαβ cells (A) and those of CD8αα and CD8αβ T cells (B) among colonic intestinal epithelial lymphocytes (IELs) gated on CD3 were determined on day 7 of DSS administration to normal chow diet (NCD) or HFD-fed mice. (C and D) Proportion of myeloid subsets. Proportions of CD11b^+^Ly6C^+^ monocytes (C) and CD11b^+^F4/80^+^ macrophages (D) in colonic epithelium on day 7 of DSS treatment were determined by flow cytometry. All values are expressed as the means ± SEM. A student’s *t*-test was used to compare values between two groups. *P < 0.05, **P < 0.01 (*n* = 5 each).

When we characterized colonic inflammation following DSS treatment, a lower proportion of TCRγδ T cells, which play a critical role in tissue repair [51] among IELs, was noted in HFD-fed DSS-treated mice compared to NCD-fed DSS-treated mice (Fig 6A). The proportions of TCRαβ T cells, important lymphoid cells among IELs controlling intestinal immune responses whose dysregulation leads to the development of colitis [52], were inversely higher in HFD-fed DSS-treated mice compared to NCD-fed DSS-treated mice (Fig 6A). We also found that the proportion of CD8αα T cells, which express homodimer of CD8α instead of conventional CD8αβ heterodimer and play a unique protective role among IELs [26], was significantly lower in HFD-fed DSS-treated mice compared to NCD-fed DSS-treated mice (Fig 6B). When myeloid cells were analyzed, the proportion of CD11b^+^ monocytes expressing Ly6C, a marker of monocytes in the peripheral blood or bone marrow, was higher in colonic epithelia of HFD-fed DSS-treated mice compared to NCD-fed counterparts (Fig 6C), suggesting the involvement of newly recruited inflammatory monocytes in this process. The proportion of resident CD11b^+^F4/80^+^ macrophages was also higher in colonic epithelia of HFD-fed DSS-treated mice (Fig 6D), suggesting the changes of both myeloid cell subsets in the same direction in DSS-induced colitis of HFD-fed mice.

However, we did not find significant differences in the proportions of pro- or anti-inflammatory cells including CD11b^+^F4/80^+^ macrophages, CD11c^+^ dendritic cells, CD4^+^Foxp3^+^ regulatory T cells or CD4^+^IFN-γ^+^ inflammatory T cells in the lamina propria between HFD-fed and NCD-fed mice treated with DSS (S1 Fig). The components of HFD encounter the epithelium before the lamina propria in the intestine, and thus HFD-induced inflammatory changes may be more prominent in colonic epithelium than in the lamina propria.

**Fig 7:**
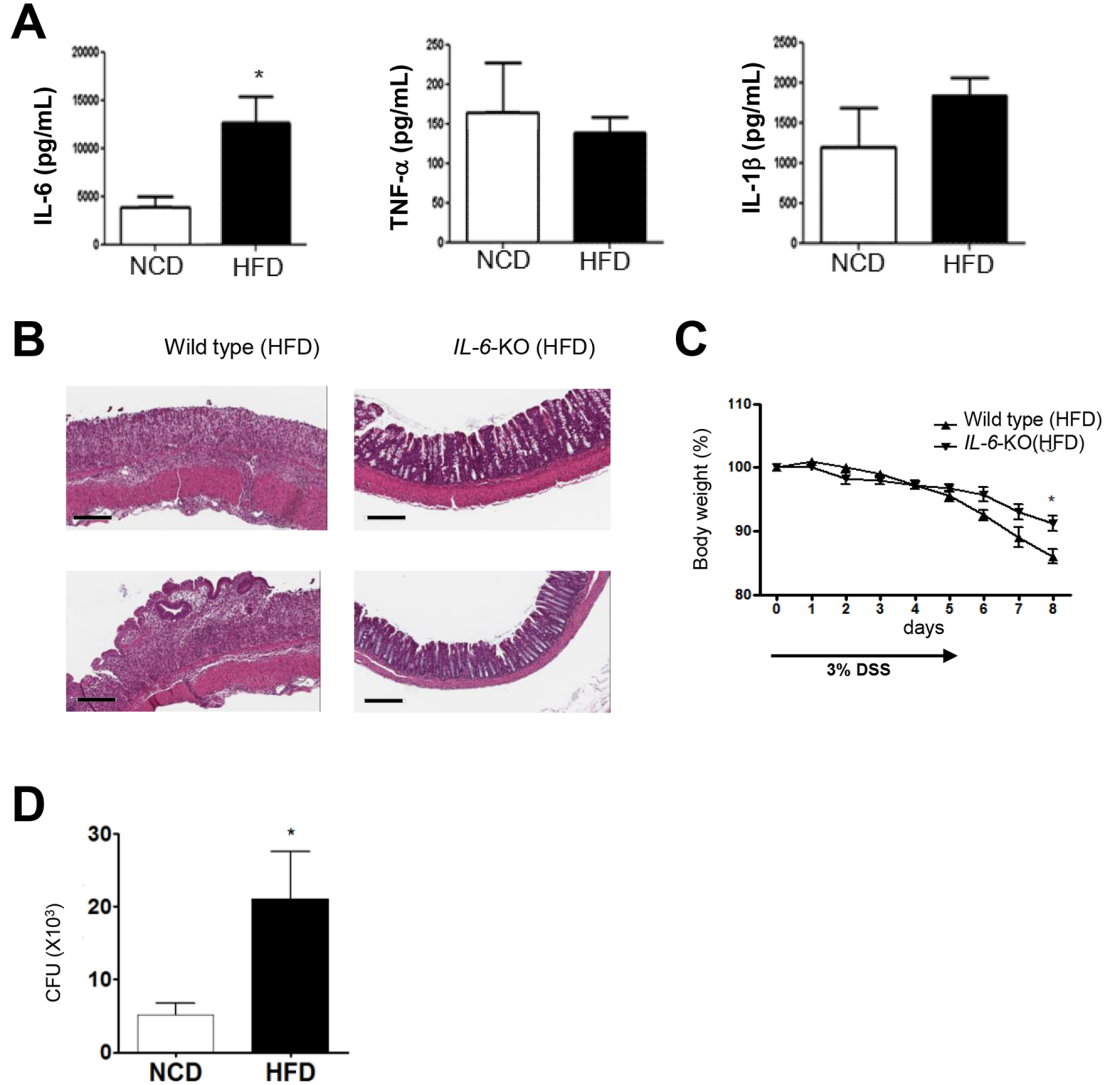
Cytokines in experimental colitis of high-fat diet (HFD)-fed mice. (A) Cytokine content in culture supernatant following overnight incubation of tissue explants was determined by ELISA on day 7 of dextran sodium sulfate (DSS) treatment. (B and C) Role of IL-6 in DSS-colitis of HFD-fed mice. DSS (3%) was administered to HFD-fed *IL-6*-knockout or wild-type mice for 5 days. Representative H&E sections of the colons on day 7 of DSS administration (B). Body weight is presented as a percentage of the initial body weight (C). (D) Bacterial translocation to the liver on day 7 of DSS treatment was quantified by colony-forming unit (CFU) counts (×1,000). All values are expressed as the means ± SEM. A student’s *t*-test was used to compare values between two groups. *P < 0.05 (*n* = 5 each). Original magnification, ×200. Scale bar, 100 μm.

When we studied cytokine production *ex vivo*, IL-6 release from the colon was significantly higher in HFD-fed DSS-treated mice compared to NCD-fed DSS-treated mice. However, the release of other inflammatory cytokines such as TNF-α and IL-1β did not differ between the two groups (Fig 7A), consistent with a previous report [53]. We further studied the role of IL-6 in DSS-colitis using *IL-6*-knockout mice. Microscopic examination revealed a marked amelioration of DSS-induced histological changes in the colons of HFD-fed *IL-6*-knockout mice compared to HFD-fed control mice (Fig 7B). Body weight loss following DSS administration was also significantly lower in HFD-fed *IL-6*-knockout mice compared to HFD-fed wild type mice (Fig 7C), suggesting that IL-6 may play a significant role in DSS-colitis of HFD-fed mice.

We also studied systemic bacterial invasion in DSS-induced colitis. Significantly increased CFU counts were observed in the liver of HFD-fed DSS-treated mice compared to NCD-fed DSS-treated mice (Fig 7D), suggesting an increased invasion of indigenous intestinal bacteria into sterile tissue through the disrupted gut barrier.

### Effects of HFD and DSS on gut microbiota

**Fig 8:**
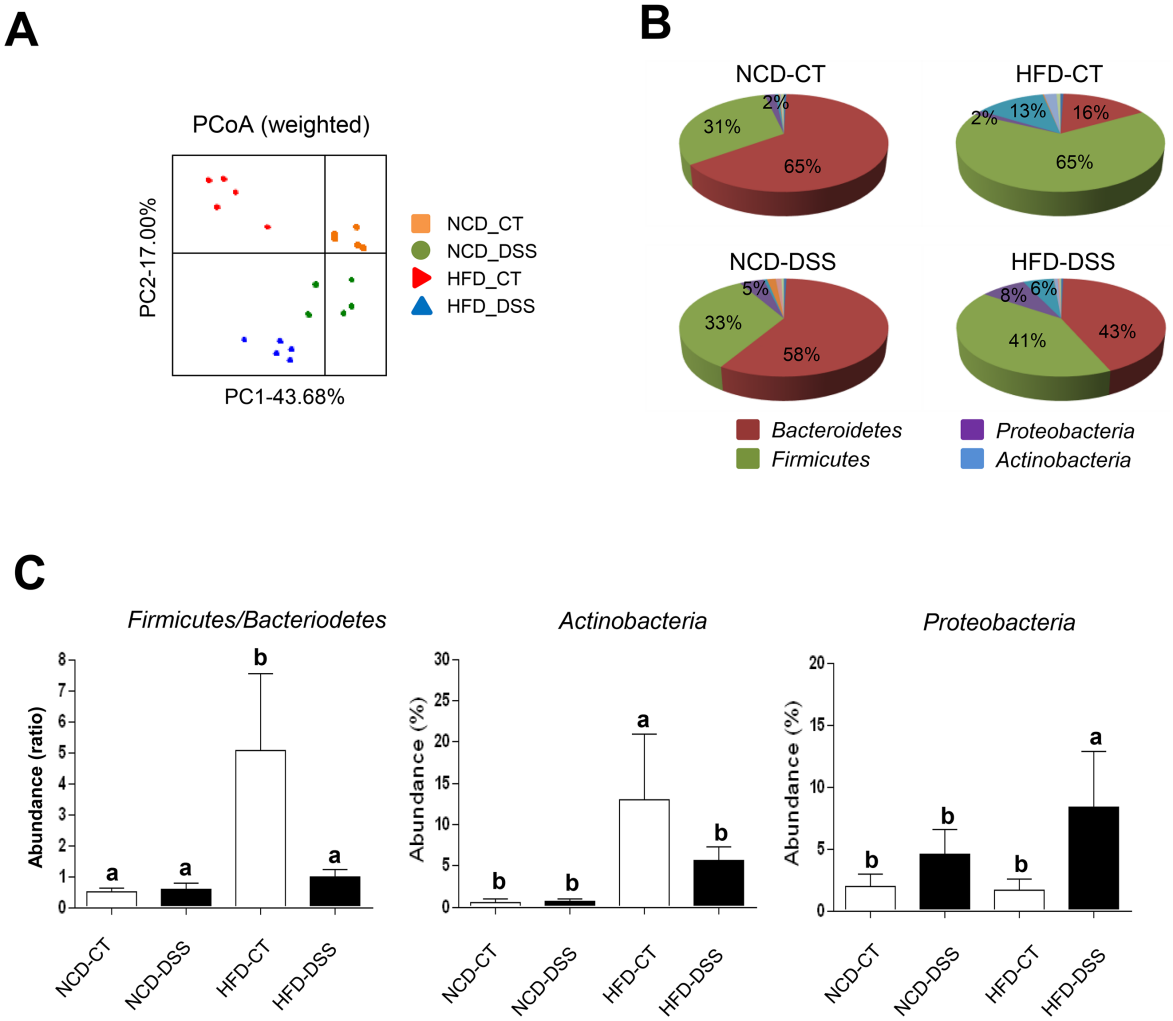
Alteration of intestinal microbiota following high-fat diet (HFD) feeding and dextran sodium sulfate (DSS) treatment. (A) Weighted principal coordinates analysis (PCoA) plots. Each spot represents one sample, and each group of mice is denoted by a different color. (B and C) Phylum-level changes of gut microbiota following HFD feeding and DSS treatment. Pie charts showing the differences in the relative abundances (%) of bacterial phyla (B). Changes of specific phyla (C). Normal chow diet (NCD)-control (CT), NCD-fed mice without DSS treatment; NCD-DSS, NCD-fed mice with DSS treatment; HFD-CT, HFD-fed mice without DSS treatment; HFD-DSS, HFD-fed mice with DSS treatment (*n* = 5 each). A one-way ANOVA was used to compare values among multiple groups. If an ANOVA test showed significant differences, the Duncan post-hoc test was used to compare two specific groups. If the Duncan test did not show a significant difference (*P* > 0.05), the two groups were labeled with the same letter over the respective bars. *P* values < 0.05 were considered statistically significant.

We next determined the effects of HFD consumption and DSS treatment on gut microbiota using 16S rRNA gene-based 454 pyrosequencing of 20 fecal samples from mice belonging to 4 different groups: NCD-fed mice without DSS treatment (NCD-CT), NCD-fed mice with DSS treatment (NCD-DSS), HFD-fed mice without DSS treatment (HFD-CT), and HFD-fed mice with DSS treatment (HFD-DSS). When the overall compositions of microbial communities in the different groups were investigated by weighted PCoA of UniFrac distance-based comparisons, a marked change in the microbial composition was noted following consumption of a HFD (Fig 8A). Furthermore, the microbial communities in HFD-DSS mice formed a cluster that was distinct from those of HFD-CT mice. In contrast, the microbial communities of NCD-CT mice were closely clustered with those of NCD-DSS mice, indicating that DSS treatment has a more profound effect on gut microbiota of HFD-fed mice compared to NCD-fed mice (Fig 8A). At the phylum level, HFD-CT mice exhibited a higher abundance of *Firmicutes* (*P* < 0.001) and a lower abundance of *Bacteroidetes* compared to NCD-CT mice (*P* < 0.001) (Fig 8B and 8C, S2 Fig), consistent with previous reports [54]. Interestingly, HFD-CT mice had a significantly higher abundance of *Actinobacteria* compared to NCD-CT mice (*P* < 0.01) (Fig 8B and 8C, S2 Fig). Analysis of the effect of DSS on the composition of gut microbiota revealed that DSS treatment induced a profound shift in HFD-fed mice, but only slight changes in NCD-fed mice (Fig 8B). Moreover, an increased abundance of *Firmicutes* relative to *Bacteroidetes* in HFD-CT mice was abrogated by DSS treatment (*P* < 0.001) (Fig 8B and 8C). While it was significantly reduced following DSS treatment, the abundance of *Actinobacteria* in HFD-DSS mice still tended to be higher than that in the NCD-DSS mice (Fig 8B and 8C). The abundance of *Proteobacteria* in HFD-fed mice was significantly increased following induction of colitis (*P* < 0.01) (Fig 8B and 8C), in line with previous reports [55].

**Fig 9:**
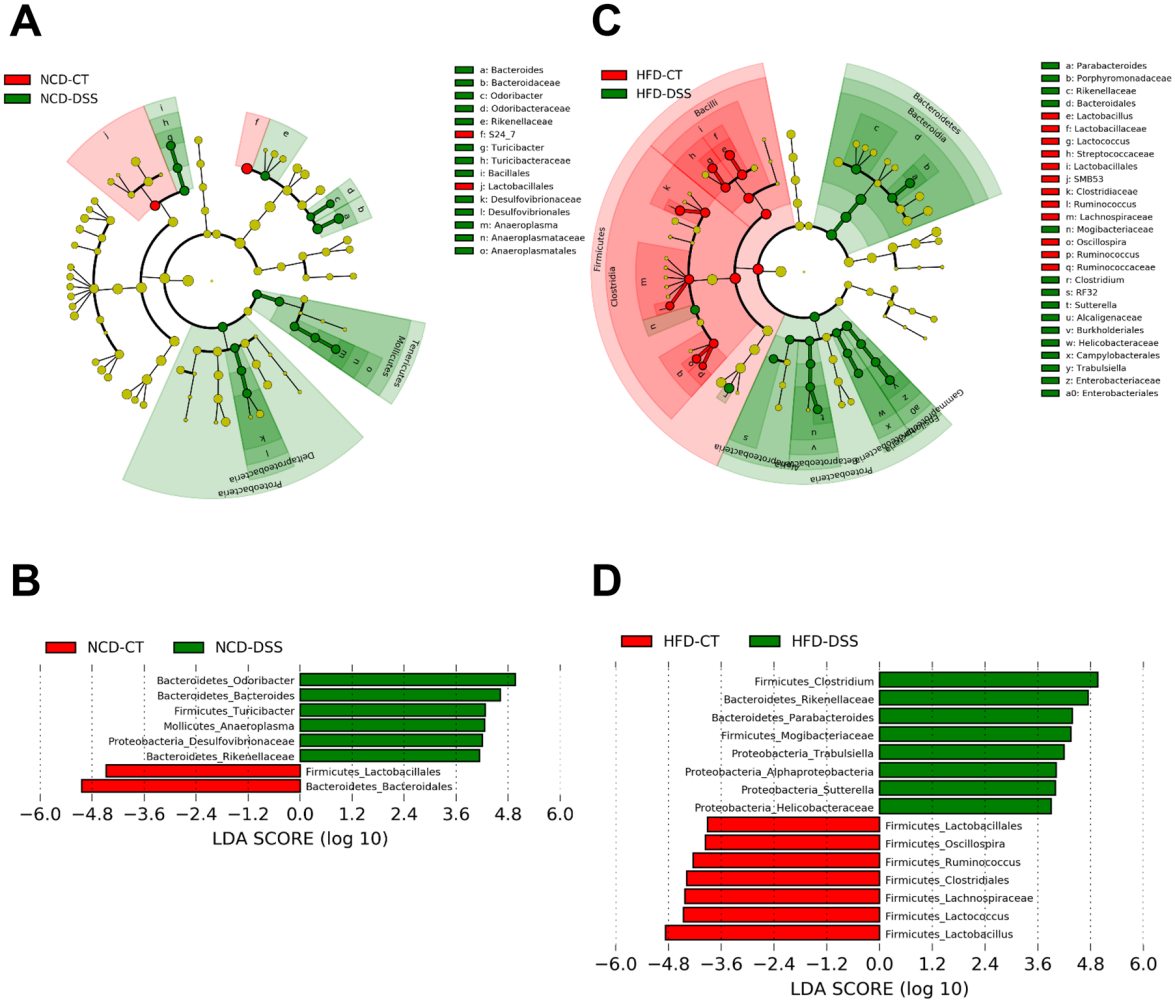
Linear discriminant analysis (LDA) effect size (LEfSe) analysis of gut microbiota changes following consumption of a high-fat diet (HFD) and dextran sodium sulfate (DSS) treatment. Effect of DSS. (A and B) Normal chow diet (NCD)-control (CT) vs. NCD-DSS mice. (C and D) HFD-CT vs. HFD-DSS mice. The phylogenetic tree and histogram show LDA scores calculated for differences in genus-level abundance between DSS-treated and -untreated mice. NCD-CT, NCD-fed mice without DSS treatment; NCD-DSS, NCD-fed mice with DSS treatment; HFD-CT, HFD-fed mice without DSS treatment; HFD-DSS, HFD-fed mice with DSS treatment (*n* = 5 each). The colors represent the group in which the indicated taxa is more abundant compared to the other group. The LDA scores of the NCD-CT and HFD-CT were negative, while those of the NCD-DSS and HFD-DSS were positive. Such negativity or positivity is determined by alphabetical order of the groups, and the absolute values of the effect size indicate the scale of the difference between 2 groups regardless of the positivity or negativity.

**Fig 10:**
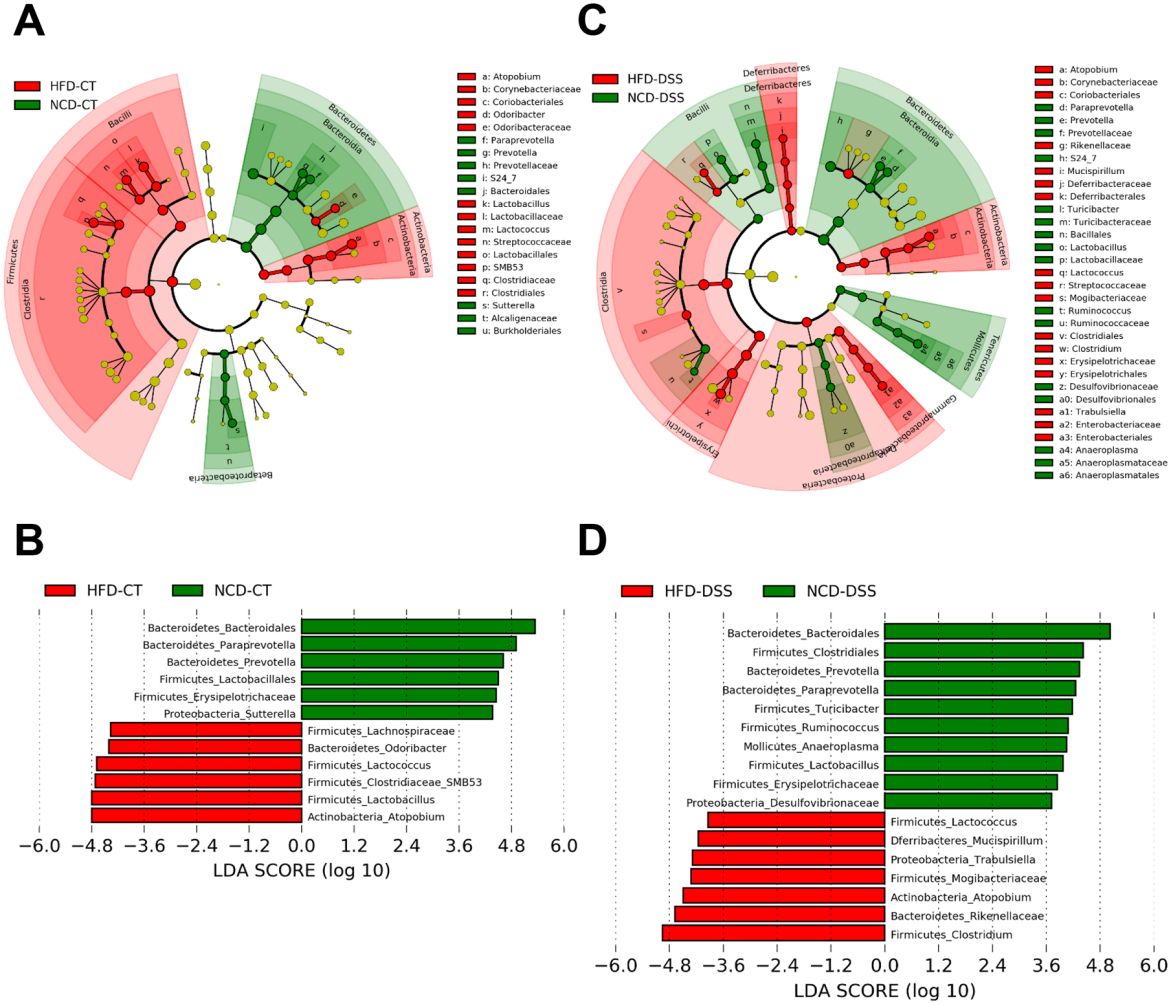
Linear discriminant analysis (LDA) effect size (LEfSe) analysis of gut microbiota changes following consumption of a high-fat diet (HFD) and dextran sodium sulfate (DSS) treatment. Effect of diet. (A and B) Normal chow diet (NCD) control-CT vs. HFD-CT mice. (C and D) NCD-DSS vs. HFD-DSS mice. The phylogenetic tree and histogram show LDA scores calculated for differences in genus-level abundance between mice fed different diets. NCD-CT, NCD-fed mice without DSS treatment; NCD-DSS, NCD-fed mice with DSS treatment; HFD-CT, HFD-fed mice without DSS treatment; HFD-DSS, HFD-fed mice with DSS treatment (*n* = 5 each). As in Fig 9, the colors represent which group the taxa was more abundant compared to the other group. The LDA scores of the HFD-CT and HFD-DSS were negative (Fig 10B), while those of the NCD-CT and NCD-DSS were positive. The absolute values of the effect size indicate the scale of the difference between 2 groups regardless of the positivity or negativity.

We next studied specific phylotypes that respond to DSS treatment using the linear discriminant analysis (LDA) effect size (LEfSe) algorithm identifying more abundant taxa in one group compared to the other group (Figs 9 and 10) [56]. Eight genus-level phylotypes were discovered as high-dimensional biomarkers discriminating gut microbiota between NCD-DSS and NCD-CT mice (Fig 9A and 9B). In the HFD groups, phylotype alterations following DSS treatment were more dramatic compared to the NCD groups (Fig 9C and 9D). Fifteen genus-level phylotypes were discovered as biomarkers discriminating gut microbiota between HFD-DSS and HFD-CT mice (Fig 9D). In particular, the levels of 3 phylotypes, *Trabulsiella*, *Sutterella*, and *Helicobacteraceae*, which belong to the phylum *Proteobacteria* and may drive colitis progression, were higher in HFD-DSS mice compared to HFD-CT mice.

Genus-level comparisons of NCD and HFD groups revealed that gut microbial community structures were markedly different them, consistent with previous studies [1,2]. When NCD and HFD groups without DSS treatment were compared (Fig 10A and 10B), 12 phylotypes were identified as key markers of distinct gut microbiota according to the different diets (Fig 10B). Interestingly, the abundance of *Atopobium* which belongs to the *Actinobacteria* phylum, showed the most significant difference between the HFD-CT and NCD-CT groups (0.5% in the NCD group vs. 12.8% in the HFD group). When NCD and HFD groups were compared after DSS treatment (Fig 10C and 10D), 17 phylotypes were identified as key markers that differentiate gut microbiota between diets (Fig 10D). Intriguingly, the abundance of *Trabulsiella* in the phylum *Protebacteria*, and *Atopobium*, which was increased in abundance in HFD-CT mice compared to NCD-CT mice, was increased in HFD-DSS mice compared to NCD-DSS mice, implying that these taxa may play a colitogenic role under HFD-fed conditions (Fig 10D). *Atopobium* and 2 of the 3 phylotypes in the phylum *Protecbacteria* (*Sutterella* and *Helicobacteraceae*) that were increased in HFD-DSS mice were detectable in healthy mice also (S3 Fig), and thus could be considered colitis-inducing pathobionts following consumption of a HFD.

### Effects of antibiotic treatment and FMT on colitis

**Fig 11:**
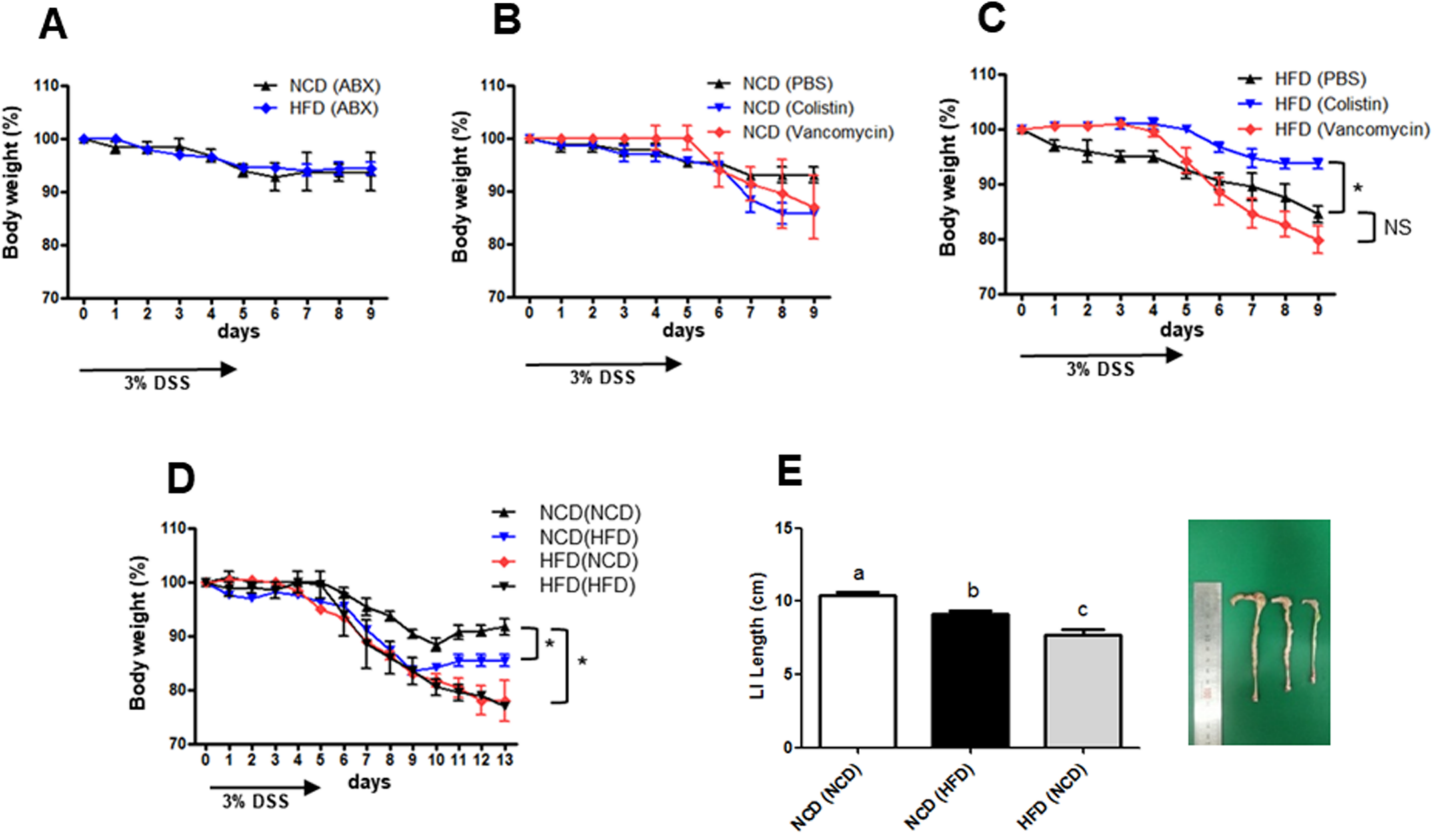
Fecal microbial transplantation (FMT) and antibiotic treatment alter colitis progression. (A-C) Antibiotic treatment. Mice were fed normal chow diet (NCD) or high-fat diet (HFD), and an antibiotic combination (A) or a single antibiotic (B and C) was administered for 8 weeks. Subsequently, DSS-colitis was induced. (D-F) Effects of FMT. NCD- or HFD-fed mice gavaged with fecal material from the same or the other group were treated with dextran sodium sulfate (DSS) and monitored for colitis development. Body weight is presented as the percentage of the initial weight (D). Colon lengths on day 13 of DSS treatment (E, left). Images of representative colons (E, right). (*n* = 5 each). *P < 0.05. All values are expressed as the means ± SEM. A student’s *t*-test was used to compare values between two groups. A one-way ANOVA was used to compare values among multiple groups. If an ANOVA test showed significant differences, the Duncan post-hoc test was used to compare two specific groups. If the Duncan test did not show a significant difference (*P* > 0.05), the two groups were labeled with the same letter over the respective bars. *P* values < 0.05 were considered statistically significant. *P < 0.05 (*n* = 5 each). NS, not significant.

We next administered a broad-spectrum antibiotic mixture to study the role of intestinal bacteria in DSS-induced colitis. The previously noted difference in body weight between the HFD-fed and NCD-fed mice following DSS administration (Fig 5A) was abrogated by antibiotics (Fig 11A), suggesting that bacterial translocation is linked to aggravated colitis in HFD-fed mice. To study the effects of depleting specific clades of gut microbiota, we used two different narrow-spectrum antibiotics, vancomycin, which depletes Gram-positive bacteria, and colistin, which depletes most Gram-negative bacteria [57]. Neither antibiotic affected the progression of DSS-induced colitis in NCD-fed mice (Fig 11B). However, colistin, but not vancomycin, ameliorated the severity of DSS-induced colitis in HFD-fed mice (Fig 11C), suggesting that Gram-negative bacteria such as strains of *Proteobacteria*, participate in the progression of experimental colitis in HFD-fed mice.

We also studied whether the altered intestinal microbiota induced by the consumption of different diets is responsible for different colitis susceptibility employing FMT technology. Mice fed NCD or HFD were gavaged with fecal material obtained from their littermates on the same diet or from mice on a different diet, resulting in 4 groups of mice: NCD-fed mice transplanted with NCD microbiota, NCD(NCD); NCD-fed mice transplanted with HFD microbiota, NCD(HFD); HFD-fed mice transplanted with NCD microbiota, HFD(NCD); and HFD-fed mice transplanted with HFD microbiota, HFD(HFD). Three weeks following FMT, these mice were untreated or treated with DSS for 5 days and monitored for colitis progression. There were no differences in the percent body weight between NCD(HFD) and NCD(NCD) mice without DSS treatment (S4 Fig). However, loss of body weight following DSS treatment was more severe in NCD(HFD) mice compared to NCD(NCD) mice (Fig 11D), which suggests more severe colitis in DSS-treated NCD(HFD) mice compared to DSS-treated NCD(NCD) mice and is likely due to transfer of colitogenic bacteria in the feces of HFD-fed mice to the NCD-fed mice. Colon length of NCD(HFD) mice after DSS treatment was also significantly reduced compared to DSS-treated NCD(NCD) mice (Fig 11E). HFD(NCD) mice treated with DSS lost more body weight and exhibited shorter colons compared to NCD(NCD) mice (Fig 11D and 11E), which is likely due to persistent colitogenic bacteria in HFD-fed mice following FMT.

## Discussion

When we examined the intestine, one of the first organs contacting dietary lipids, we observed a marked reduction in Paneth cell area and function following consumption of a HFD, accompanied by reduced expression of AMPs in HFD-fed mice [58]. The decrease in Paneth cell area may have been caused by cell death due to lipid injury, as was shown by the increased number of 7-AAD-stained CD24^+^ cells in mice fed HFD. In terms of function, bactericidal activity of Paneth cell culture supernatant from mice fed HFD was also reduced. In this study, we did not further investigate other mechanisms that may affect Paneth cell number, such as the generation or differentiation of Paneth cells. A recent study reported that consumption of a HFD increased the number of intestinal stem cells by 50% but reduced Paneth cells by 23%, suggesting that a HFD may impair Paneth cell differentiation [59]. Thus, impaired differentiation of intestinal stem cells to Paneth cells and increased Paneth cell death could contribute to the reduced Paneth cell number and area observed in HFD-fed mice.

Paneth cell dysfunction is linked to IBDs such as Crohn’s disease [5], and the incidence of IBD is increased with consumption of a Western diet [12,13]. We therefore studied the impact of HFD-induced Paneth cell dysfunction in IBD using a DSS-colitis model. We found that the severity of DSS-induced colitis was augmented by consumption of a HFD, suggesting that fatty acids or other lipid metabolites in a Western diet may be environmental risk factors for IBD.

Individual strains or specific combinations of microorganisms have also been implicated in the pathogenesis of colitis [14,60]. In this study, we found that consumption of a HFD with or without enteropathic chemicals resulted in a significantly increased population of *Atopobium* sp., which has been reported to be associated with Crohn’s disease [61] and colorectal cancer [62]. However, we did not identify specific species among the *Atopobium* genus that were increased in HFD-fed mice, precluding further studies on the role of *Atopobium*. Some strains belonging to the *Proteobacteria* phylum are also colitogenic [55,60]. In this study, the proportions of *Proteobacteria* phylotypes such as *Trabulsiella*, *Sutterella*, and *Helicobacteraceae*, which are related to gastrointestinal pathology [61,63], were increased in HFD-fed DSS-treated mice. Moreover, colistin treatment targeting Gram-negative bacteria attenuated colitis in HFD-fed mice, suggesting that strains of *Proteobacteria* play a crucial role in the colitis of HFD-fed mice. FMT from HFD-fed mice also significantly augmented the progression of colitis in NCD-fed mice, implying that altered microbial ecology induced by consumption of a HFD increases susceptibility to colitogenic stimuli.

Our study has some limitations. First, the unphysiologically high fat content in our HFD (60%) could have caused artifactual effects that are not seen in human subjects consuming Western diets (~35% fat). Second, a major difference between purified HFD and regular NCD, in addition to dietary lipid composition and concentration, includes the absence of soluble fiber in HFD [64]. While normal chow diet (NCD) consists of 13% fat, 25% protein, 62% carbohydrate in calories and contains crude fiber, HFD consist of 60% fat, 20% protein, 20% carbohydrate in calories and contains only insoluble fiber, like cellulose. Soluble fiber is known to be important in gut health. So, lack of soluble fiber in HFD might have contributed to the intestinal dysfunction and have confounded our experiments. Third, our FMT may also have technical limitations because it was not conducted in a germ-free setting or with antibiotic pretreatment. Residual host microbiota and possible incomplete transplantation of donor microbiota to the recipients may explain the less dramatic loss of body weight following DSS administration to NCD(HFD) mice compared to HFD(HFD) mice. Although FMT is usually performed in germ-free or antibiotic-treated mice, a pyrosequencing study showed a close resemblance of recipients’ microbiota to donors’ only after FMT without antibiotics [37]. We believe that some key species were successfully transplanted following FMT from HFD-fed mice to NCD-fed mice, which can explain the significant increase in body weight loss after DSS treatment in NCD(HFD) mice compared to NCD(NCD) mice. Fourth, the changes of Paneth cells observed in this investigation might not be specific for Paneth cells. As shown in Figs 2 and 3, death of crypts cells and decreased number of goblet cells were observed in mice fed HFD, suggesting changes similar to those of Paneth cells might occur in other types of intestinal cells.

Our findings demonstrate that consumption of a HFD impairs gut barrier function maintained by AMPs and mucin predisposing the subject to the development of IBD, while it is not clear whether dietary fat or altered composition of other constituents such as fiber in HFD is the main culprit leading to such impairment. Furthermore, alteration of gut microbiota by consumption of a HFD appears to contribute to an increased susceptibility to colitis. Thus, modulation of the viability and function of Paneth cells or goblet cells and that of microbiota by probiotics, prebiotics, or host-microbial metabolites [65] may have therapeutic potential against IBD, particularly in cases associated with high fat intake.

## Supporting information

S1 Fig Flow cytometric analysis of cells in colonic lamina propria of high-fat diet (HFD)-fed mice treated with dextran sodium sulfate (DSS). The proportions of CD11b^+^F4/80^+^ macrophages, CD11c^+^ dendritic cells (DCs), CD4^+^Foxp3^+^ Tregs and CD4^+^IFN-γ^+^ inflammatory T cells in colonic lamina propria of HFD- or normal chow diet (NCD)-fed mice on day 7 of DSS treatment are shown.
(TIF)

S2 Fig Phylum-level changes in fecal microbiota following consumption of a high-fat diet (HFD) and treatment with dextran sodium sulfate (DSS). Fecal microbiota compositions in 20 different mice. Normal chow diet (NCD)-control (CT), NCD-fed mice without DSS treatment; NCD-DSS, NCD-fed mice with DSS treatment; HFD-CT, HFD-fed mice without DSS treatment; HFD-DSS, HFD-fed mice with DSS treatment (*n* = 5 each).
(TIF)

S3 Fig Relative abundance of pathobionts. Differences in the relative abundances (%) of *Atopobium*, *Sutterella*, *Trabulsiella*, and *Helicobacteraceae* in normal chow diet (NCD)- or high-fat diet (HFD)-fed mice before and after dextran sodium sulfate (DSS) treatment. *Atopobium*, *Sutterella*, and *Helicobacteraceae* were detected by pyrosequencing even in healthy mice, suggesting they are pathobionts. NCD-CT, NCD-fed mice without DSS treatment; NCD-DSS, NCD-fed mice with DSS treatment; HFD-CT, HFD-fed mice without DSS treatment; HFD-DSS, HFD-fed mice with DSS treatment (*n* = 5 each).
(TIF)

S4 Fig Changes in percent body weight during 2-week observation period without dextran sodium sulfate (DSS) treatment following fecal microbial transplantation (FMT). FMT was conducted for 3 weeks in mice fed a high fat diet (HFD) or normal chow diet (NCD) for 15 weeks. Body weight is presented as the percentage of the initial weight. NCD(NCD), NCD-fed mice transplanted with NCD microbiota; NCD(HFD), NCD-fed mice transplanted with HFD microbiota; HFD(NCD), HFD-fed mice transplanted with NCD microbiota; HFD(HFD), HFD-fed mice transplanted with HFD microbiota (*n* = 5 each).
(TIF)
