# Supplementary figures and images for: Obesogenic diet-induced gut barrier dysfunction and pathobiont expansion aggravate experimental colitis
Source: PLoS One. 2017 Nov 6;12(11):e0187515. doi: 10.1371/journal.pone.0187515 (PMC5673181; doi:10.1371/journal.pone.0187515)

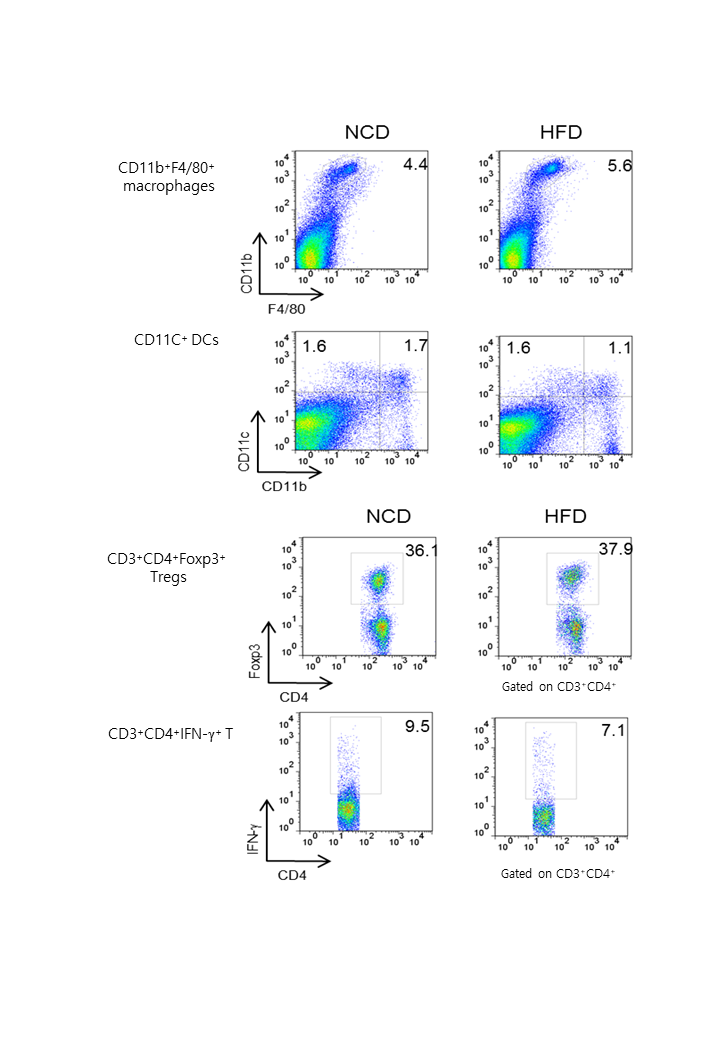

Supplement: S1 Fig — The proportions of CD11b+F4/80+ macrophages, CD11c+ dendritic cells (DCs), CD4+Foxp3+ Tregs and CD4+IFN-γ+ inflammatory T cells in colonic lamina propria of HFD- or normal chow diet (NCD)-fed mice on day 7 of DSS treatment are shown. (TIF) [file pone.0187515.s001.tif]

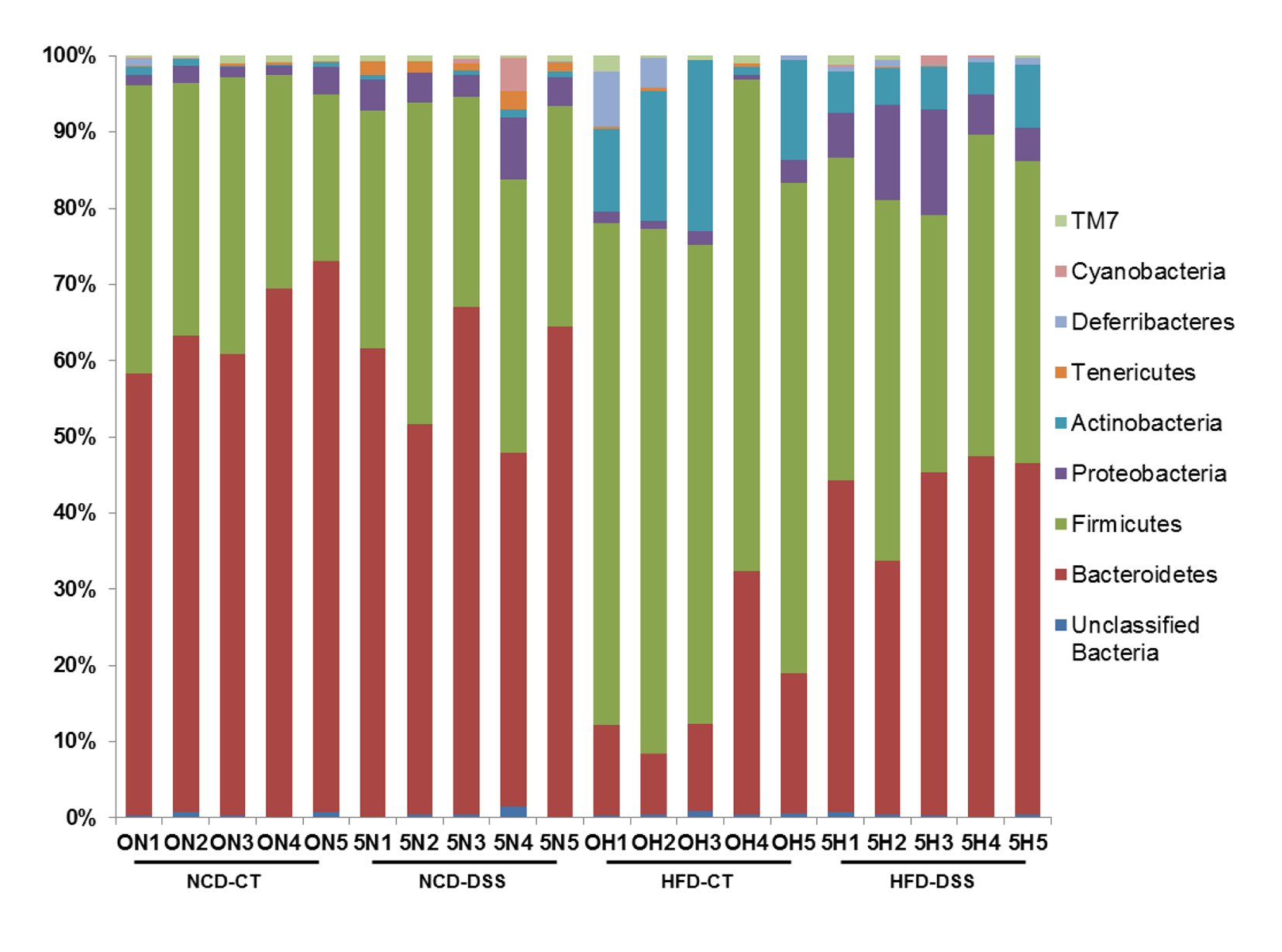

Supplement: S2 Fig — Fecal microbiota compositions in 20 different mice. Normal chow diet (NCD)-control (CT), NCD-fed mice without DSS treatment; NCD-DSS, NCD-fed mice with DSS treatment; HFD-CT, HFD-fed mice without DSS treatment; HFD-DSS, HFD-fed mice with DSS treatment (n = 5 each). (TIF) [file pone.0187515.s002.tif]

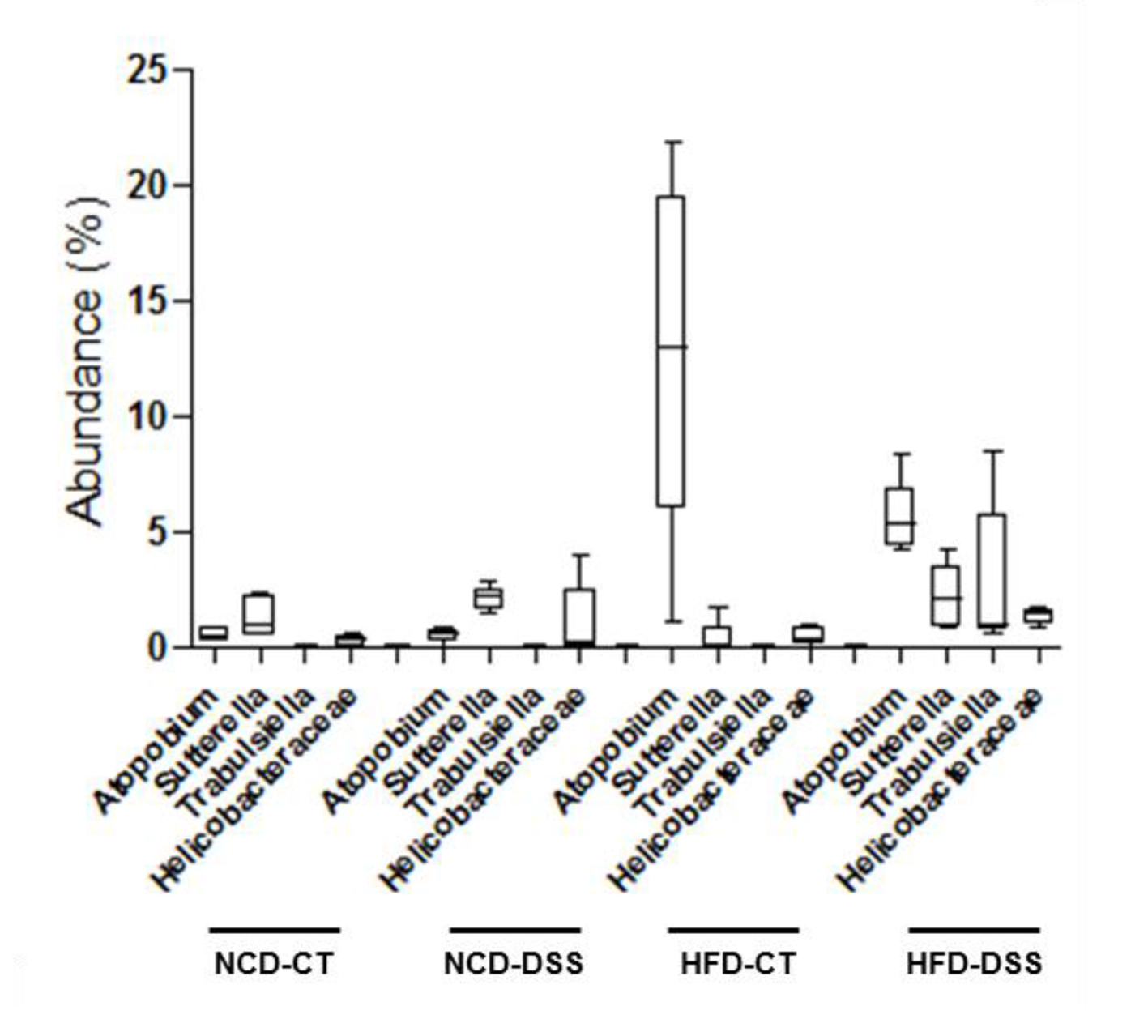

Supplement: S3 Fig — Differences in the relative abundances (%) of Atopobium, Sutterella, Trabulsiella, and Helicobacteraceae in normal chow diet (NCD)- or high-fat diet (HFD)-fed mice before and after dextran sodium sulfate (DSS) treatment. Atopobium, Sutterella, and Helicobacteraceae were detected by pyrosequencing even in healthy mice, suggesting they are pathobionts. NCD-CT, NCD-fed mice without DSS treatment; NCD-DSS, NCD-fed mice with DSS treatment; HFD-CT, HFD-fed mice without DSS treatment; HFD-DSS, HFD-fed mice with DSS treatment (n = 5 each). (TIF) [file pone.0187515.s003.tif]

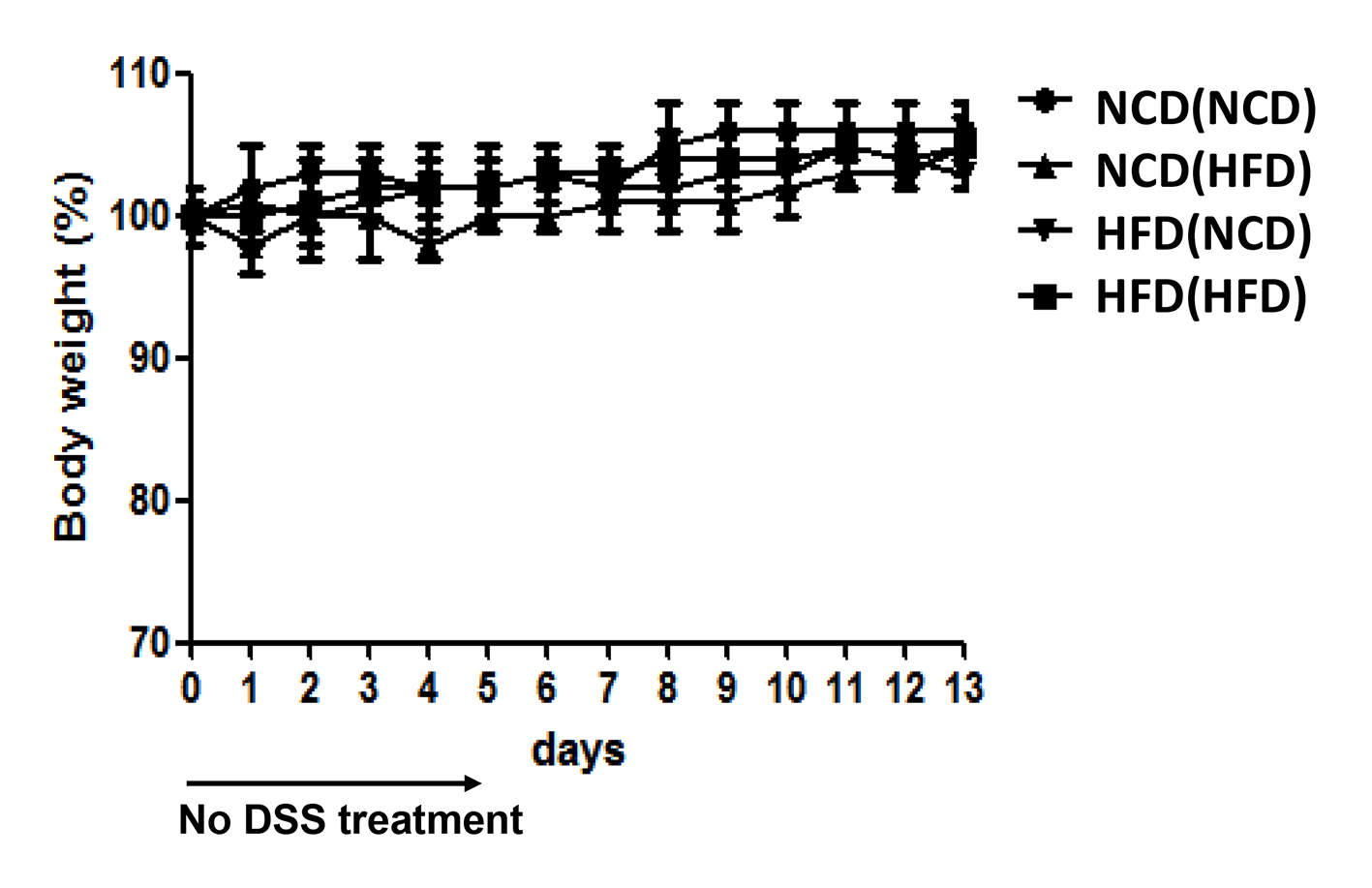

Supplement: S4 Fig — FMT was conducted for 3 weeks in mice fed a high fat diet (HFD) or normal chow diet (NCD) for 15 weeks. Body weight is presented as the percentage of the initial weight. NCD(NCD), NCD-fed mice transplanted with NCD microbiota; NCD(HFD), NCD-fed mice transplanted with HFD microbiota; HFD(NCD), HFD-fed mice transplanted with NCD microbiota; HFD(HFD), HFD-fed mice transplanted with HFD microbiota (n = 5 each). (TIF) [file pone.0187515.s004.tif]
